# Glucosafe 2—A new tool for nutritional management and insulin-therapy in the intensive care unit: Randomized controlled study (the Glucosafe 2 protocol)

**DOI:** 10.1371/journal.pone.0316624

**Published:** 2025-03-04

**Authors:** Aude de Watteville, Ulrike Pielmeier, Mariagrazia Di Marco, Angèle Gayet-Ageron, Nils Siegenthaler, Nicolas Parel, Hannah Wozniak, Steve Primmaz, Jérôme Pugin, Steen Andreassen, Claudia Paula Heidegger

**Affiliations:** 1 Division of Intensive Care, Department of Acute Care Medicine (DMA), Geneva University Hospitals, Geneva, Switzerland; 2 Clinical Nutrition, Department of Medicine (DME), Geneva University Hospitals, Geneva, Switzerland; 3 Respiratory and Critical Care Group (R-Care), Aalborg University, Aalborg, Denmark; 4 Clinical Investigation Unit, Clinical Research Center, Geneva University Hospital, Geneva, Switzerland; 5 Methodological Support Unit, Clinical Research Center, Geneva University Hospital, Geneva, Switzerland; 6 Department of Anaesthesiology, Pharmacology, Intensive Care and Emergency Medicine, Faculty of Medicine, University of Geneva, Geneva, Switzerland; Sapienza University of Rome: Universita degli Studi di Roma La Sapienza, ITALY

## Abstract

**Background:**

Patients admitted to the Intensive Care Unit (ICU) can experience significant fluctuations in blood glucose levels, even if they do not have a history of diabetes. Such variations may arise from multiple causes and are part of the adaptative stress-response to critical illness. To support their nutritional needs, these patients might also need parenteral feeding. Glucose and metabolic fluctuations can lead to serious consequences, including increased infection rates, loss in protein and muscle mass and increased morbi-mortality. This justifies precise and constant monitoring. The management of insulin therapy and nutritional therapy strongly impacts the outcomes of critically ill patients. Glucosafe 2 (GS2) is an innovative medical device designed to address these needs. It offers real-time recommendations to healthcare professionals regarding blood glucose control and nutritional inputs among ICU patients. The goal is to ensure that blood glucose levels remain within the desired range of targeted values, and consequently to minimize the risk of both hypo- and hyperglycemia.

**Method and design:**

This study is an unblinded randomized controlled study with: (1) the intervention group, which uses the GS2 device for nutritional therapy and blood glucose advice until discharge from the ICU or up until 15 days after study enrolment; (2) the control group, which uses standard care according to local ICU protocols. We also collected data of a third historical control group using retrospective data from a sample of ICU patients exposed to the standard of care 2 years before the start of the prospective trial; it aims first to validate the predictive accuracy of the GS2 model before the start of the prospective parts and to interpret the existence of possible bias by assessing the potential cross-contamination effects between intervention and control group, due to the fact that caregivers can take more care of patients in the control group, which will dilute the effect of GS2. We planned to enrol 71 patients per group (total =  213 patients). The primary objective is to compare the time spent within a predetermined range of glycemia (5.0 – 8.5 mmol/l) between the intervention group (GS2) and the control group (standard local ICU protocols).

**Discussion:**

This study aims to evaluate the performance and safety of the GS2 medical device software to monitor and guide blood glucose management and nutritional therapy in critically ill patients in comparison to current standard of care. If proven successful, GS2 could be used to optimize nutritional and blood glucose management. The clinical data gathered from this study will also contribute to the Clinical Evaluation Report (CER), a regulatory document that provides an assessment of the clinical safety and performance of a medical device throughout its intended lifecycle. GS2 has the potential to optimize the quality of nutritional and blood glucose management and improve compliance with international guidelines.

**Trial registration:**

ClinicalTrials.gov, NCT03890432, Registered on 26 March 2019

## 1. Introduction

The management of blood glucose (BG) by insulin therapy and nutritional therapy strongly influences the outcomes of critically ill patients. Hypo- or hyperglycaemia are associated with higher mortality, especially in non-diabetic patients [1,2]. An association between high blood glucose (BG) variability and increased infection rate and mortality has also been observed [3,4]. Early studies on BG control focused on lowering BG level and on maintaining them within a range of near-normal BG concentrations. These studies have shown to be able to reduce up to 45% of ICU/in-hospital mortality rates [4–7]. Some later clinical trials failed to reproduce the improvement of in ICU/hospital mortality obtained in the early studies [8,9]. The causes behind this discrepancy are still being discussed, with one reason being the failure to adhere to the clinical trial protocols [10,11] and thus to reach the intended BG target. Noteworthy, the rate of hypoglycemic episodes observed in these studies was high [8,9].

In parallel to improving BG control, a proper management of the nutritional therapy can also result in lower morbidity and mortality rates in the ICU [12–14]. It is known that inadequate nutritional therapy contributes to protein loss and muscle wasting, leading to ICU-acquired weakness [15–17] and a further increase of the ICU and post-ICU morbi-mortality [18–20]. ICU acquired weakness has been shown to be associated with increased mortality, persistent functional impairment, and cognitive impairment [6].

International professional societies (e.g., European Society for Clinical Nutrition; American Society for Parenteral and Enteral Nutrition; German Society for Nutritional Medicine) have published evidence-based guidelines for glycaemic control and nutritional therapy of ICU patients [21–23]. Implementation of these guidelines in an ICU setting proves to be quite challenging. In fact, the level of compliance with local guidelines is limited, with only around 50% compliance observed for BG control [24,25]. Regarding nutritional therapy, patients who stayed in the ICU for over 72 hours have been found to receive less than 60% of their estimated energy expenditure on average [26,27].

Glucosafe 2 (GS2) is a decision support system intended to help caregivers in achieving optimised BG control and nutritional therapy. It is based on a mathematical model of the glucose-insulin metabolism that integrates the following data: body mass (BM), age, sex, presence and type of diabetes, renal replacement therapy, serial blood glucose measurements, insulin therapy, non-nutritional calories, and the nutrition support.

Based on these data, GS2 calculates the patient’s insulin sensitivity and provides patient-specific recommendations for adapting insulin therapy and nutritional therapy and gives users the appropriate advice. The advice is in the format of a set of pump rates, one for the infusion rate for the insulin pump (Ul/h) and rates (ml/h) for each infusion pump delivering calories and/or proteins to the patient. This presentation of GS2 advice emphasises that insulin and nutrition therapy both affect blood glucose levels. Therefore, insulin and nutrition advices should be given at the same time. It is hoped that this format will help to reduce the lack of compliance seen in some of the studies mentioned above. For this reason, one of the aims of the trial is to measure caregiver compliance.

GS2 is designed to carefully control blood glucose levels, reduce glycaemic variability and decrease harmful hyper- or hypoglycaemic events. In addition, it allows for better coverage of energy and protein targets and, to some degree, compensation for cumulative caloric and protein deficits.

## 2. Objectives

The aim of the study is to validate the efficacy and safety of GS2 as a bedside tool compared to the standard of care. The goal is to improve the management of nutritional therapy and BG control in critically ill patients.

The primary objective is to compare the time spent in a predetermined glycaemic range (5.0 – 8.5 mmol/l) between two randomised groups of patients, all presenting with hyperglycaemia (defined as presenting one BG measurement >  10 mmol/l or two BG measurements >  8.5 mmol/l) and having an expected ICU length of stay of ≥  72h:

1) An intervention group with insulin therapy and nutritional therapy guided by GS2.
2) A control group with insulin therapy and nutritional therapy guided by local protocols.

The secondary objectives of the study are to improve energy and protein intake, in line with international recommendations, and to reduce BG variability by the use of GS2 compared to standard care. We will also analyse a third historical control group to validate the predictive accuracy and to investigate the potential bias from possible cross-contamination, where the actions of caregivers in the control group could attenuate the effect of GS2.

The main safety objective is to avoid hypoglycemia and to reduce hyperglycaemia thanky to the guidance of glycemia control by GS2.

Compliance will be assessed from the differences between the advice provided by GS2 and the insulin, calories and proteins actually provided to the patient.

## 3. Methods

### 3.1. Justification for the design of the clinical investigation

Fewer episodes of hyper- and hypoglycaemia are associated with lower mortality rates and adequate nutritional therapy is associated with a reduction of ICU-acquired weakness and of morbidity both in the ICU and post-ICU. As described above, the aim of the study is to assess whether GS2 can safely and efficaciously reduce hyper- and hypoglycaemia and align the nutritional therapy with nutritional guidelines.

#### 3.1.1.Study design.

The GS2 study is a single centre, investigator-initiated, unblinded two-arms randomised controlled trial.

- **Intervention group:** Use of GS2 until the patient is discharged from the ICU, or for a maximal follow-up period of 15 days, or until the patient starts eating (voluntary feeding). Number of patients: 71
- **Control group**: Standard care according to local ICU protocols (Geneva-HUG). Follow-up is performed until discharge from the ICU, or for a maximal follow-up period of 15 days, or until the patient starts eating (voluntary feeding). Number of patients: 71

Recruitment started in January 2023 and planned to be achieved in December 2024.

A third historical control group will also be analysed. The aim is to validate the predictive accuracy of the GS2 model prior to the prospective phase of the study and to assess potential bias due to possible cross-contamination. This is important as GS2 users may care for patients in both groups and use the same procedures in the control group as in the intervention group, thereby attenuating the effect of GS2.

**Historical control group:** Retrospective data from the computerised records of 71 ICU- patients (2 years before the start of the prospective phase) who underwent standard care regarding nutrition support and blood glucose control. Follow-up is performed until discharged from the ICU of for a maximal follow-up period of 15 days, or until the patient starts eating (voluntary feeding). Data extraction for the historical control group was performed on the 16th of June 2022, using the same selection criteria as for the intervention and control groups*.* Number of patients: 71

#### 3.1.2. Explanation for the choice of the comparator.

In the control and the historical control groups, patients are treated in accordance with the local protocols described below. As mentioned before, the historical control group has 2 purposes:

1) To validate the predictive accuracy of the GS2 model before the start of the prospective randomized part of the study
2) To assess the size of potential cross-over effects from the GS2 advice, which may influence the clinicians’ treatment of the patients in the control group. The 2-year period prior to the start of the prospective phase was chosen to avoid the period of the COVID-19 epidemic and to have the patients most representative of those normally admitted to the ICU. There has been no change in standard BG control management or nutritional therapy since then.

### 3.2. Approvals and registration

The GS2 study was approved by the competent Ethics Committee (Commission Cantonale d’Ethique de la Recherche, CCER) and the Competent Authority (Swissmedic) on the 21^st^ of December 2021 (BASEC: 2021-D0034). Current version of the Declaration of Helsinki [28], the European Regulation on medical devices 2017/745 (MDR) [29], the Norms ISO14155 [30] and ISO14971 [31], the ICH-guidelines of Good Clinical Practice (GCP) [32] as applicable, the Swiss Human Research Act (HRA) [33] and its Ordinances are followed. This study is registered on the International Clinical Trial Registry website: www.clinicaltrial.gov (ID: NCT03890432).

### 3.3. Setting

The study will be conducted in the ICU of the Geneva University Hospitals. It is a mixed medical and surgical unit, with a total number of 32 beds. On average, 1500 patients are admitted each year. The following strategy will ensure that enough participants are reached to achieve the target sample size. All patients admitted to the ICU will be screened from Monday to Friday by one of the co-investigators of the study. Screening will be performed from patients’ admission until day 5 of their ICU stay. If eligibility criteria are met, participants (or their legal representatives) will be approached with explanation about the study as well as to provide them with the information sheet and the consent form. If the participant is unable to consent and no legal representative could be reached, a physician not associated with the study is called to safeguard the interests of the person concerned (art 30 HRA) [33]. The enrolment of the patient in the study can be made when the independent physician authorization is obtained. This authorization form must be signed by the independent physician and an investigator of the study and will be retained as part of the study records. The patients’ (or their legal representatives’) consents will then be sought retrospectively.

### 3.4. Participants

Subjects fulfilling all the following inclusion criteria are eligible for the study.

All patients ≥  18 years old admitted to the ICU with

- Expected length of stay ≥  72h after inclusion in the study
- At least one BG measurement ≥ 10 mmol/l or two BG measurements ≥  8.5 mmol/l
- Informed Consent signed by the subject/ legal representative, except for patients in the historical control group for whom no consent form is required

The presence of any of the following exclusion criteria will lead to the exclusion of the patient:

- Pregnancy or breast feeding * 
- Diabetic ketoacidosis or hyperosmolar state
- Oral feeding, as we cannot accurately estimate the amount absorbed
- Fulminant hepatic failure
- Medically contraindicated for the administration of rapid-acting insulin by intravenous (IV) infusion or IV injection

*A blood pregnancy test (blood beta-hCG) will be performed in all women of childbearing potential before entering the study.

### 3.5. Randomisation

Randomisation will be performed using a computerized random number generator (secuTrial®) after validation of all eligibility criteria and operated by an independent statistician. Randomization (1:1) will be stratified on the Acute Physiologic Assessment and Chronic Health Evaluation II score (APACHE II) (mild: < 15/moderate: 15-20/severe: > 20) and on the diabetic status (diabetic/non-diabetic patients). Six randomization lists using permuted block sizes of 4, 6, or 8 methods will be used. Concealment of allocation will be guaranteed by the automatic computer-centralised generation of the randomisation list.

### 3.6. Blinding

This is an unblinded trial as the clinical and biological assessments will be directly driven by the monitoring provided by the medical device or the standard of care procedures. The statistician will be blinded to the allocation throughout the study to avoid any information bias in the interpretation of data.

### 3.7. Timeline

**Fig 1:**
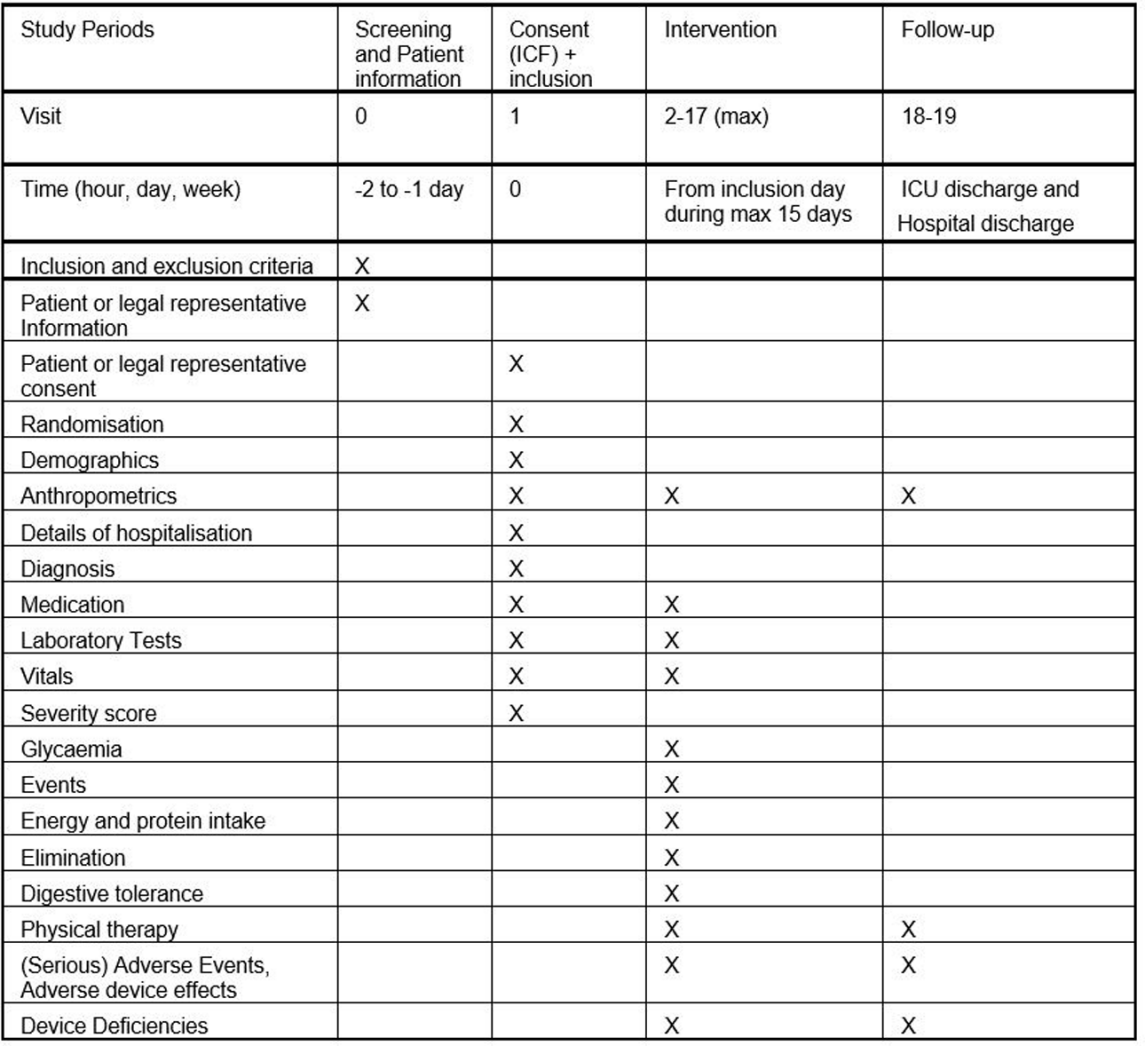
Study schedule.

All patients will be followed from their inclusion in the study until ICU discharge, with a maximum of 15 days follow-up, or until oral feeding is resumed. The timeline is similar for the 3 groups (Fig 1).

### 3.8. Intervention

#### 3.8.1. Glucosafe 2 medical device.

**Fig 2:**
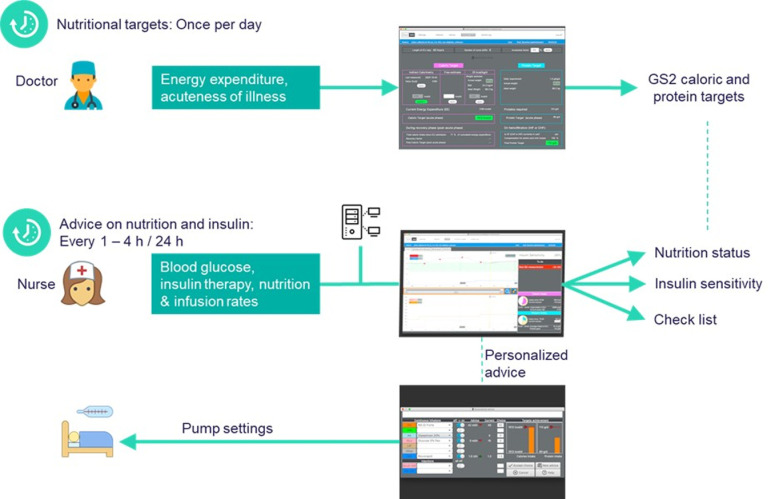
Glucosafe 2 workflow.

GS2 version 1.0 is accessible on all bed-side computers in the ICU and linked to the electronic patient management system, from which it automatically retrieves all clinical data relevant to decision support. During morning rounds, physicians check the energy and protein targets and adjust them, if necessary. Every 1 to 4 hours, depending on the stability of the BG level (timing specified by GS2 in the “To do list”), the nurse in charge of the patient measures BG and enters the result in the electronic patient management system (EPMS). These data are automatically imported into GS2 by the EPMS and used for the calculation of insulin sensitivity and for the BG prediction. If necessary, GS2 advises the caregiver to change the insulin therapy and/or the nutritional therapy. GS2 workflow is shown in Fig 2.

Nutritional targets are set once every day by the physicians in charge. The energy expenditure (EE) is first estimated as 25 kcal/kg/day. Indirect calorimetry (IC) is performed, if achievable every 72 hours and the result is directly included in GS2. As soon as an IC measurement is available, the result will be imported from the EPMS and will then become the estimated EE. GS2 then suggests a caloric target as a percentage of EE. This percentage is called the “Acuteness Factor” and is gradually increased to 100% during the first days of the ICU stay. The physician can freely adjust the Acuteness Factor, if needed, in case of a new infection for example.

Similarly, the protein target is set as a daily requirement of 1.3 g/kg/day, multiplied with the Acuteness Factor. In case of special requirement, the physician can either increase or decrease the protein target.

GS2 provides the caregiver with advice on insulin and nutritional therapy management whenever requested by the caregiver, typically after every new BG measurement. GS2 advice has the format of a set of pump rates, one for the infusion rate for the insulin pump (Ul/h) and rates (ml/h) for each of the infusion pumps delivering calories and/or proteins to the patient. The nutrition pump rates are calculated from the caloric and protein content of the nutrition products chosen for the patient, such that the advice satisfies the patient’s caloric and protein targets. The advice on nutrition may fall short of the caloric and protein targets in situations where the patient’s hyperglycaemia cannot be controlled by insulin alone. The user can either follow the advice, modify it according to the clinical situation, or refuse it. If the user modifies or refuses the advice, the user also has the possibility to give a comment to explain the reason. The GS2 advice is accompanied by comments as a help to understand the given advice.

**3.8.1.1. Training and strategies to improve compliance:** All caregivers at the Geneva ICU will participate in training sessions before the beginning of the study. They will also receive a summary brochure with the main information, and a demonstration video will be available at any time. During the trial, a clinical investigator will help the caregiver and one-to-one training sessions will be given, if needed. Moreover, GS2 contains a “To do list” to remind the user of upcoming tasks, e.g., the time of the next BG measurement and comments are also provided in connection with the advice to help the user to understand it better.

#### 3.8.2. Standard of care.

**Fig 3:**
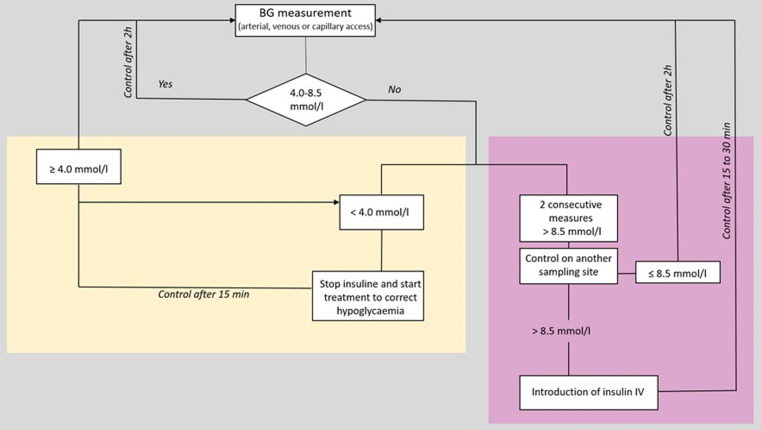
Algorithm of the local blood glucose control protocol. BG: blood glucose; IV: intravenous.

**Fig 4:**
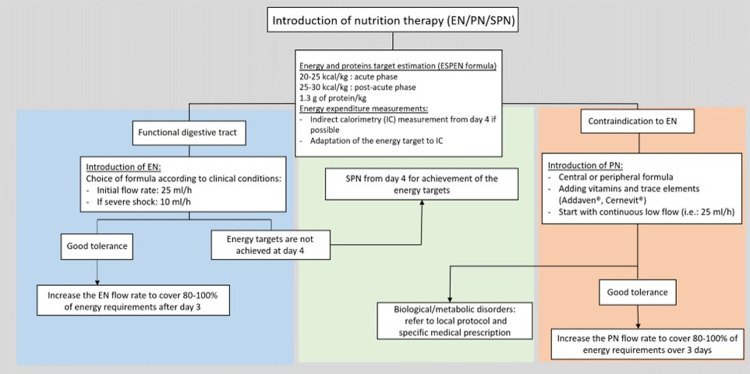
Algorithm of the local nutrition therapy protocol. EN: enteral nutrition; PN: parenteral nutrition; SPN: supplemental parenteral nutrition.

Current local protocols for glycaemia control and nutrition management will be used in the control group and in the historical control group for comparison. Algorithms of the local blood glucose control and nutrition therapy protocols are shown in Fig 3 and Fig 4 below.

Glycaemic control protocol. After ICU admission, a BG measurement is performed. If glycaemia is in the accepted range (5.0-8.5 mmol/l) or spontaneously normal (4.0 to 5.5 mmol/l), no correction is proposed. In this case, a BG control should be done every 2 hours.

In the case where two consecutive BG measurements are >  8.5 mmol/l, a new control is proposed in another sampling site. If the BG measurement is still 8.5 mmol/l, an IV insulin-therapy should be initiated following the local protocol. A further BG control should be done within the next 15-30 minutes until BG stabilizes. Then, BG controls should be done every 2 hours.

If a BG measurement is lower than 4 mmol/l, insulin-therapy should be stopped (if in progress) and a correction can be performed. A new BG measurement is done within 15 minutes. If the following BG measurement is still lower than 4 mmol/l, a new correction is done with G40%. Once the BG is greater than 4 mmol/l, BG measurements are spaced to every 30 minutes. Then, when the BG is greater than 5 mmol/l, BG control measurements are done every 2 hours.

Nutritional therapy protocol. An estimation of the caloric target is performed at the admission in the ICU. If the patient stays more than 4 days and the indirect calorimetry measurement is achievable (PEEP max. 10 and FIO2 max. 70%), the measurement is performed, and the caloric target is adjusted. If the gastrointestinal tract is functional and there is no contraindication to enteral nutrition (EN), this is started on the first day with an appropriate product depending on the pathology (products used in clinical routine, which may be isocaloric, hypercaloric or hyperproteinic depending on the situations). The initial rate is of 25 ml/h or 10 ml/h for a patient with severe shock (can be adapted according to tolerance). The 80-100% of the energy target need to be achieved at the end of day 3. If the energy target (80-100%) is not reached at day 4, supplemental parenteral nutrition (SPN) is added.

If EN is contraindicated, parenteral nutrition (PN) can be initiated in a progressive way. It can be either by central venous access or by peripheral venous access. Vitamins and trace elements should be supplemented with 1 vial of Addaven^®^ and 1 vial of Cernevit^®^. Glutamine supplements can also be added on a case-by-case basis. If tolerance is good, PN is progressively increased to achieve 80-100% of the energy target by the end of day 3.

### 3.9. Data collection

To ensure the quality of data, the principal investigator (PI) will train the study staff on all important study-related aspects and will ensure that the study is performed according to the protocol, guidelines for good clinical practice (ICH-GCP) and regulations.

Data will be automatically imported from the electronic patient management system to GS2, and further data, generated during the use of the system, that pertain to study outcomes will be extracted automatically from Glucosafe’s database and are kept in a secured storage. Other data will be entered by a member of the research team directly into the secuTrial^®^ platform. The secuTrial^®^ platform allows full auditing and traceability of modifications. Solely the research team and the PI will have access to the platform after a secure authentication. Members of the research team authorized by the PI to enter the data in the eCRF will be listed on the delegation log.

All data will be pseudonymised in the secuTrial^®^ platform and in the Glucosafe extractions. The subject identification list will be secured by a password and will be kept in a secured storage of the Geneva University Hospitals. Data management will be performed by the Clinical Investigation Unit (Clinical Research Center) of Geneva University Hospitals.

### 3.10. Assessment of outcomes

All outcomes will be assessed per patient and per cohort

Primary outcome

Time-in-target (range: 5.0 to 8.5 mmol/l) per patient and in the cohort:

- All the BG measurement will be recorded with the time of the measurement in the eCRF. Then the time spent in the target (5.0 to 8.5 mmol/l) will be calculated in percentage for each patient and for each of the patient groups.

Secondary outcomes:

- Safety: From the BG measurements extracted the number and percentage of mild ( ≤ 3.2 mmol/l) and severe ( ≤ 2.2 mmol/l) hypoglycaemic events will be calculated. Additionally, and only in the intervention group, the number and percentage of mild and severe hypoglycaemic events due to non-compliance with Glucosafe’s advice will be calculated.
- Glycaemic control:
  - Time to normalize blood glucose (defined as time from study enrolment to the time of the first of two consecutive BG measurements ≤  8.5 mmol/l)
  - Time/ Episodes of hyperglycaemia ( > 8.5 mmol/l) before/ after normalization
  - Time/ Episodes of hypoglycaemia/ hyperglycaemia due to non-compliance with GS2 advice (intervention group only)
  - Mean and standard deviation of BG
  - Maximum of daily BG difference
- Nutrition management, that will be calculated from the caloric and protein daily intake as well as the caloric and protein daily targets:
  - Daily caloric intake in percent of daily caloric target, also as average over study days
  - Daily protein intake in percent of daily protein target, also as average over study days
  - Cumulated caloric and protein deficits on the last study day
- User acceptance (intervention group only):
  - Number of episodes where a new BG measurement is not followed up within 30 minutes by a user request for GS2 advice
  - Number of episodes where pumps were not set within 30 min according to a GS2 advice accepted by the nurse (nurse did not notice, was busy, forgot or misinterpreted advice)
  - Number of advices given by GS2 which were accepted, accepted with modification and rejected
- Comparison of the workload between intervention and control groups:
  - Difference in frequency of BG measurements (mean and standard deviation)
  - Difference in frequency of adjustments of insulin and nutrition pump settings
- Glucosafe 2-Model accuracy:
  - The accuracy of the model prediction of the BG level varies with the prediction time: the model prediction error is the difference between the ‘predicted’ and the ‘measured’ BG level.
- Insulin sensitivity profiles according to time in the ICU, ICU survival, and BG measurements:
  - The insulin sensitivity profile is as a graphical representation of the model-calculated insulin sensitivity (a numerical patient parameter) and the time point, for which the insulin sensitivity parameter was calculated.

### 3.11. Safety

Device deficiencies, adverse events (AE), and serious adverse events (SAE) are fully investigated and documented in the safety report form and appropriate case report form during the entire study period (from patient’s informed consent until the end of the ICU stay or the end of the 15 days of follow up, whatever comes first) [ISO 14155]. Documentation includes date of the event, treatment, resolution, assessment of seriousness and causal relationship to device and/or study procedure.

Reporting of events to the Competent Ethics Committee (CEC) and to Swissmedic will be subcontracted to the pharmacovigilance team of the Clinical Investigation Unit of Geneva University Hospitals.

The following events are to be reported to the CEC and to the competent authority (CA) promptly (Art. 33 ClinO-MD) [34]:

a. any serious adverse event which has a causal relation with the medical device or procedure/test method or where a causal relation is probable or possible (SADE);
b. any device deficiency (DD) which, in the absence of appropriate measures or intervention or in less favourable circumstances, could have led to serious adverse events (DD with SADE potential);
c. any new information relating to an event already notified under points (a) and (b).

### 3.12. Data safety monitoring committee

A data safety monitoring committee is not planned for this study. The purpose of the study is the “safety and efficacy” of a new medical device. The study will be discontinued if the following issues arise: ethical concerns; insufficient recruitment of subjects; if five patients in the intervention group have two severe hypoglycaemias (≤2.2 mmol/l); ten device deficiencies that prevent the use of Glucosafe 2 (GS2) for 4 hours; changes in accepted clinical practice that make it unwise to continue the study; early evidence of benefit or harm of the experimental intervention.

### 3.13. Study monitoring

For quality control purposes, the study site is visited on site by a qualified monitor. An initiation visit will be conducted on site to ensure that all study materials and infrastructure are ready for the beginning of the study. Follow-up visits are scheduled for every 10 patients enrolled.

### 3.14. Statistical analysis

#### 3.14.1. Power calculation.

Based on routinely collected data in our ICU, we estimated that a mean 68% (SD 21%) of critically ill patients are in the time-in-target. Based on an expert consensus, we have considered an absolute mean improvement of 10% of BG time-in-target as clinically important (going from mean 68% to 78% time-in-target), we would need 71 patients per arm (power of 80%, two-sided α-error =  5%). We predefined the same number of patients in the historical control arm.

#### 3.14.2. Primary analysis.

The primary analysis will be done using a mixed linear regression model with the repeated mean proportions time-in-target collected during the ICU stay as the dependent variable, the study arm as the independent variable adjusted for the two randomisation stratification variables, the APACHE II categories (mild/moderate/severe) and the diabetic status (diabetic/non-diabetic patients), and the patient ID as the random factor on the intercept. We will conclude to superiority of the GS2 intervention, compared to the control group, if the time in target is significantly higher (p < 0.05).

#### 3.14.3. Secondary analysis.

For all secondary continuous and repeated outcomes, we will apply the same model, mixed linear regression model with the repeated measurements as the dependent variable, the study arm as the independent variable adjusted for the two randomisation stratification variables, and the patient ID as the random factor on the intercept. For binary and repeated outcomes (protein goal achievement, caloric goal achievement), we will apply mixed logistic regression model (or if data are over-dispersed, mixed negative binomial regression models), with the binary repeated outcomes as the dependent variable, the study arms as main independent variable, and adjustment for the two randomisation stratification variables. For count and repeated outcomes, if data are over-dispersed, we will apply mixed negative binomial regression models, with the count repeated outcomes as the dependent variable, the study arms as main independent variable, and adjustment for the two randomisation stratification variables.

### 3.15. Publication and dissemination policy

The results of the study are published in the form of one or more articles in international English-language journals. Results, both positive and negative and inconclusive, will be published.

## 4. Discussion

Critically ill patients, with their high metabolic instability and different illness phases, require special attention and care [13,17,35]. Blood glucose control and prevention of glycemic excessive variability are crucial in improving clinical outcomes [5,6,8]. Coupling the optimization of nutritional therapy with a good glycemic control could benefit critically ill patients by providing a patient-centered approach. The combination of optimized nutritional therapy with good BG control could be beneficial for critically ill patients, as it represents a patient-centered approach and is adapted to the needs of patients in different phases of illness.

To our knowledge, the GS2 medical device software is the first tool to consider BG management and nutritional therapy as a unified metabolic approach, rather than using separate tools or methods.

The outcomes of the study were designed to assess safety (frequency of hypo- and hyperglycemic episodes), efficacy (time in band for BG, glycemic variability, and achievement of nutritional goals) and compliance (nurse acceptance of GS2 advice). If this clinical trial demonstrates that GS2 can achieve safety, efficacy, and compliance, GS2 could be used in studies investigating clinically relevant outcomes such as mortality and morbidity in intensive care patients. In the light of the Medical Device Regulation (MDR), there is an increasing emphasis on collecting robust clinical data for medical devices. For products such as Glucosafe 2, which currently does not have a CE marking, conducting a rigorous clinical study is essential to gather substantial evidence of safety and efficacy. This is particularly important in order to meet the strict requirements of the MDR and pave the way for approval.

Therefore, tools like GS2 can significantly improve patient care by being tailored to individual needs. This potential improvement based on robust clinical evidence, is in line with the overall objectives of the MDR to ensure both patient safety and the delivery of innovative medical solutions.

### Roles and responsibilities

The sponsor of the study is the Geneva University Hospitals, represented by Claudia Heidegger, MD, PhD. Claudia Heidegger, deputy head physician at the Division of the Intensive Care, is also the principal investigator of the study. The manufacturer of the Glucosafe 2 software is the Aalborg University, a public academic institution in Denmark.

Their contact information can be found below:

- Sponsor/Principal Investigator

Claudia-Paula Heidegger, MD, PhD, Deputy head physician

Division of Intensive Care, Geneva University Hospitals

Rue Gabrielle-Perret-Gentil 4, 1211 Geneva 14, Switzerland

Phone: + 41-22 372 74 40

Email: claudia-paula.heidegger@hug.ch

- As Project manager for the Manufacturer

Ulrike Pielmeier, PhD, Biomedical engineer

 Dept. of Health Science and Technology, Aalborg University

 Selma Lagerløfs Vej 249, 12.03.049

 9260 Gistrup, Denmark

 Phone: + 45 23 83 17 14

 Email: upiel@hst.aau.dk

## Supporting information

S1 Data SPIRIT Fillable checklist.
(PDF)

S2 Data Clinical Investigation Plan.
(PDF)
