# Supplementary material for: Glucosafe 2—A new tool for nutritional management and insulin-therapy in the intensive care unit: Randomized controlled study (the Glucosafe 2 protocol)
Source: PLoS One. 2025 Mar 4;20(3):e0316624. doi: 10.1371/journal.pone.0316624 (PMC11878923; doi:10.1371/journal.pone.0316624)
Supplement: S2 Data — Clinical Investigation Plan. (PDF) [file pone.0316624.s002.pdf]

GLUCOSAFE 2 – A NEW TOOL FOR NUTRITIONAL MANAGEMENT AND INSULIN-THERAPY IN THE INTENSIVE CARE UNIT: RANDOMIZED CONTROLLED STUDY

Clinical Investigation Plan (CIP)

Type of investigation:	Randomized controlled study
Categorisation:	Category C2 according to Art 6 ClinO-MD
Registration:	Name of the study: Glucosafe 2<br>ClinicalTrial.gov registration number: NCT03890432
Identifier:	GLUCOSAFE 2 Clinical Investigation plan<br>GS2-08-2021
Principal Investigator and Sponsor, or Sponsor-Investigator:	Dr Claudia Paula Heidegger, Associate Medical Officer,<br>Department of Acute Medicine (DMA)/ Division of Intensive Care, Geneva University Hospitals (HUG).
Sponsor representative (if the Sponsor is not located in Switzerland)	Dr Claudia- Paula Heidegger, Representative of the Geneva University Hospital (HUG).
Medical Device:	Name of the device: Glucosafe 2<br>Type: class IIb: Decision support software medical device giving advice to physicians and nurses for the management of nutrition and insulin therapy.<br>GMDN code: 61087
CIP Version and Date:	Version 1.3: 28.02.2023

Table of Contents

SYNOPSIS **5**
ABBREVIATIONS **12**
INVESTIGATION SCHEDULE **14**
1. INVESTIGATION ADMINISTRATIVE STRUCTURE **15**
1.1 Sponsor, Sponsor-Investigator 15
1.2 Principal Investigator(s) 15
1.3 Statistician ("Biostatistician") 15
1.4 Laboratory 15
1.5 Monitoring institution 15
1.6 Data Safety Monitoring Committee 15
1.7 Any other relevant Committee, Person, Organisation, Institution 16
2. ETHICAL AND REGULATORY ASPECTS **16**
2.1 Registration of the investigation 16
2.2 Categorisation of the investigation 16
2.3 Competent Ethics Committee (CEC) 16
2.3.1 Reporting duties to the Competent Ethics Committee 16
2.4 Competent Authorities (CA) 16
2.4.1 Reporting duties to the competent authorities 16
2.5 Ethical Conduct of the Investigation 16
2.6 Declaration of interests 16
2.7 Patient Information and Informed Consent 17
2.7.1 Request for authorization under Article 34 of the HRA 17
2.8 Subject privacy and confidentiality 18
2.9 Early termination of the investigation 18
2.10 Clinical investigation plan amendments 18
2.11 Deviation from the Clinical Investigation Plan 18
3. BACKGROUND AND RATIONALE **19**
3.1 Background and Rationale for the clinical investigation 19
3.2 Identification and description of the Investigational Medical Device 19
3.3 Preclinical Evidence for GS2 20
3.3.1 Preclinical testing of Glucosafe 20
3.3.2 Conclusion from the preclinical evidence for Glucosafe 21
3.3.3 Clinical evidence for Glucosafe 21
3.3.4 Summary of the clinical evidence for Glucosafe 23
3.4 Clinical Evidence to Date 23
3.5 Justification for the design of the clinical investigation 24
3.6 Explanation for choice of comparator 24
3.7 Risk evaluation (Risk-to-Benefits rationale) 24
3.8 Justification of the choice of the investigation population 25
4. CLINICAL INVESTIGATION OBJECTIVES **25**
4.1 Overall Objective 25
4.2 Primary Objective 25
4.3 Secondary Objectives 25

4.4 Safety Objectives 25
5. CLINICAL INVESTIGATION OUTCOMES 26
5.1 Primary Outcome 26
5.2 Secondary Outcomes 26
5.3 Other Outcomes of Interest 26
5.4 Safety Outcomes 26
6. CLINICAL INVESTIGATION DESIGN 27
6.1 General clinical investigation design and justification of design 27
6.2 Methods for minimising bias 27
6.2.1 Randomisation 27
6.2.2 Blinding procedures 27
6.2.3 Other methods for minimising bias 27
6.3 Unblinding Procedures (Code break) 27
7. CLINICAL INVESTIGATION POPULATION 28
7.1 Eligibility criteria 28
7.2 Recruitment and screening 28
7.2.1 Intervention and control groups 28
7.2.2 Historical control group 28
7.3 Assignment to investigation groups 28
7.4 Criteria for withdrawal / discontinuation of subjects 28
8. CLINICAL INVESTIGATION INTERVENTION 29
8.1 Identity of the medical device under investigation 29
8.1.1 Experimental Intervention (medical device) 29
8.1.2 Control Intervention (standard/routine/comparator) 32
8.1.3 Labelling and Supply (re-supply) 34
8.1.4 Storage Conditions 34
8.2 Discontinuation or modifications of the intervention 34
8.3 Compliance with clinical investigation intervention 34
8.4 Data Collection and Follow-up for withdrawn subjects 34
8.5 Clinical investigation specific preventive measures 34
8.6 Concomitant Interventions (treatments) 34
8.7 Medical Device Accountability 34
8.8 Return, Analysis or Destruction of the Medical Device 35
9. CLINICAL INVESTIGATION ASSESSMENTS 36
9.1 Clinical investigation flow chart(s) / table of clinical investigation procedures and assessments 36
9.2 Assessments of outcomes 37
9.2.1 Assessment of primary outcome 37
9.2.2 Assessment of secondary outcomes 37
9.2.3 Assessment of other outcomes of interest 37
9.2.4 Assessment of safety outcomes 38
9.2.5 Assessments in subjects who prematurely stop the clinical investigation 39
9.2.6 Follow-up of the subjects after the regular termination of the clinical investigation 39
9.3 Procedures at each visit 39
9.3.1 Screening visit 39
9.3.2 Inclusion visit 39

9.3.3 Daily visit 40
9.3.4 Upon ICU leave visit 40
10. SAFETY 40
10.1 Definition and Assessment of (Serious) Adverse Events and other safety related events 40
10.2 Adverse events categorization 41
10.3 Documentation and reporting in Medical Device Category C clinical investigations 41
10.3.1 Foreseeable adverse events 42
10.3.2 Reporting of (Serious) Adverse Events, device deficiencies, and other safety related events 43
10.3.3 Follow-up of (Serious) Adverse Events 45
11. STATISTICAL METHODS 46
11.1 Hypothesis 46
11.2 Determination of Sample Size 46
11.3 Statistical criteria of termination of the investigation 46
11.4 Planned Analyses 46
11.4.1 Datasets to be analysed, analysis populations 46
11.4.2 Primary Analysis 46
11.4.3 Secondary Analyses 46
11.4.4 Interim analyses 46
11.4.5 Deviation(s) from the original statistical plan 47
11.5 Handling of missing data and drop-outs 47
12. QUALITY ASSURANCE AND CONTROL 47
12.1 Data handling and record keeping / archiving 47
12.1.1 Case Report Forms 47
12.1.2 Specification of source data and source documents 47
12.1.3 Record keeping / archiving 48
12.1.4 Archiving of essential clinical investigation documents 48
12.2 Data management 48
12.2.1 Data Management System 48
12.2.2 Data security, access and back-up 48
12.2.3 Analysis and archiving 49
12.3 Monitoring 49
12.4 Audits and Inspections 49
12.5 Confidentiality, Data Protection 49
13. PUBLICATION AND DISSEMINATION POLICY 50
14. FUNDING AND SUPPORT 50
14.1 Funding 50
14.2 Other Support 50
15. INSURANCE 50
16. REFERENCES 51
17. APPENDICES ..... ERREUR ! SIGNET NON DEFINI.

SYNOPSIS

**Sponsor / Sponsor-Investigator**	**Dr. Claudia-Paula Heidegger,** Representative of the Geneva University Hospitals (HUG).
**Title:**	GLUCOSAFE 2– A new tool for nutritional management and insulin therapy in the intensive care unit: Randomized controlled study
**Short title / Investigation ID:**	GLUCOSAFE 2/ GS2-08-2021
**Clinical Investigation Plan, version and date:**	Version 1.3: 28.02.2023
**Registration:**	Name of the study registry: ClinicalTrial.gov<br>Registration number: NCT03890432
**Category and its rationale:**	Clinical category C2 trial with an investigational medical device. This study is testing the effectiveness and the safety of the Glucosafe 2 software. The Glucosafe system will provide guidance for intensive care unit (ICU) physicians and nurses as to the modification of insulin infusion pump settings and nutritional support in order to adapt to the clinical situation at the time of intervention. The software is, however, not directly linked to the insulin and nutrition pumps and all changes remain the responsibility of the caregivers.
**Name of the MD, Unique Device Identification (UDI), name of the manufacturer**	Name of the device: Glucosafe 2<br>Type: Class IIb: Decision support software medical device giving advice to physicians and nurses for the management of nutrition and insulin therapy.<br>GMDN code:61087<br>Name of the manufacturer: Aalborg University, Danemark
**Stage of development:**	Phase II - validation of effectiveness and adverse effects
**Background and rationale:**	The survival and the outcomes of critically ill patients are strongly influenced by the management of insulin therapy and the nutritional support. All improvements in these care processes and specific therapeutic interventions result in a decreased ICU morbidity and mortality rate. In this context, experts' guidelines emphasize a personalized management for each patient including individualized nutritional therapy, effective glycaemia control and early mobilization. The Glucosafe 2 software is based on a mathematical model of the glucose-insulin metabolism. This model integrates the following data: body weight (BW), height, age, gender, type of diabetes, renal replacement therapy, time of ICU admission, serial blood glucose (BG) measurements, insulin therapy and the nutrition support (including non-nutritional calories). Based on these data, Glucosafe 2 is able to calculate the individual insulin sensitivity and to recommend the dose of insulin and the protein and energy targets required for each ICU patient according to its catabolic state. This model should allow a meticulous glycaemia control and a diminution of glycaemia variability as well as the reduction of deleterious hyper- or hypoglycaemia events. For patients where insulin sensitivity is so low that insulin alone cannot eliminate hyperglycaemia, Glucosafe 2 may suggest to decrease nutritional support. To reduce caloric debt, which is a major cause of post-ICU weakness, Glucosafe 2 will attempt to increase the nutrition support whenever tolerated by the patients. In addition, Glucosafe 2 provides information to supplement nutritional proteins by complementary amino acid perfusions in order to reach the protein target.

**Objective(s):**	The study aims to validate the effectiveness and the safety of the new Glucosafe 2 bedside tool. The goal is to improve the management of nutrition support and the glycaemia control for the critically ill patients during their ICU stay.<br>The primary objective is to compare the time spent in a predetermined glycaemia range (5.0 – 8.5 mmol/l) between three groups of patients, all having hyperglycaemia and an expected length of stay in the ICU of ≥ 72h:<br>1) Intervention group managed with nutrition support and/or insulin infusion pumps adjusted according to advice given by Glucosafe 2;<br>2) Control group receiving *Standard Care* and<br>3) Historic control group receiving *Standard Care*.<br>The secondary objectives are to bring the energy and protein intake closer to the targets of international recommendations and to reduce the variability of blood glucose.<br>The main safety objective is to avoid hypoglycemia events and reduce hyperglycaemia.

**Outcome(s):**	**Primary outcome:**<br>Time-in-target (BG in range: 5.0 to 8.5 mmol/l) per patient and in the cohort.<br><br>**Other outcomes:**<br><u>Concerning safety:</u><br>Hypoglycaemia<br>- Overall number and percentage of mild (≤ 3.2 mmol/l), and severe (≤ 2.2 mmol/l) hypoglycaemic events per patient and in the cohort<br>- Number and percentage of mild (≤ 3.2 mmol/l), and severe (≤ 2.2 mmol/l) hypoglycaemia events due to non-compliance (only in intervention arm)<br><br>Hyperglycaemia (> 8.5 mmol/l)<br>- Time to normalize blood glucose (2 values between 5.0 and 8.5 mmol/l as indicator for normalization)<br>- Percentage of time in the ICU with hyperglycaemia (BG > 8.5 mmol/l) before and after normalization per patient and in the cohort<br>- Percentage of time in the ICU with hyperglycaemia (BG > 8.5 mmol/l) before and after normalization per patient and in the cohort due to non-compliance (only in intervention arm)<br>- Number of hyperglycaemic episodes after normalization per patient and in the cohort<br>- Number of hyperglycaemic episodes after normalization per patient and in the cohort due to non-compliance (only in intervention arm)<br><br>Non-compliance (only in intervention arm)<br>- Number of episodes per patient and in the cohort where a BG measurement is not followed up within 30 minutes by a request for Glucosafe 2 advice.<br>- Number of episodes per patient and in the cohort where pumps were not set within 30 min according to a Glucosafe 2 advice accepted by the nurse (nurse did not notice, was busy, forgot, or misinterpreted advice = true errors)<br>- Number of advices given by GS2 which were accepted, accepted with modification, or rejected<br><br><u>Concerning effectiveness:</u><br><br>Achievement of nutritional goals.<br>- The protein goal is met (e.g. 80-100% of accumulated target)<br>- The caloric goal is met (e.g. 80-100% of accumulated target)<br>- Energy debt at the end of the stay<br>- Protein debt at the end of the stay<br>Workload:<br>- Frequency of BG measurements per patient and in the cohort<br>- Frequency of adjustments of insulin and nutrition pump settings<br>Glycaemic “variability”:<br>- Mean and standard deviation (SD) of BG<br>- Maximum of daily BG difference<br>Prediction of blood glucose:<br>Prediction error as a function of time elapsed since last BG measurement, per patient, per cohort.

**Design:**	Randomized controlled study with an intervention group, a control group; a historical control group will also be studied:<br>- **Intervention group**: Glucosafe 2 is used until the patient is discharged from the ICU or for a maximal follow-up of 15 days or when he/she starts eating (voluntary feeding). Number of patients: 71<br>- **Control group**: Standard care according to local ICU protocols (Geneva-HUG). The maximal follow-up is of 15 days or when he/she starts eating (voluntary feeding). Number of patients: 71.<br>- **Historical control group:** retrospective data from a random sample of 71 patients exposed to the standard care regarding nutrition support and glycaemic control and recruited before the beginning of the pilot study in order to minimize the “cross-over” effects and to confirm the predictive accuracy of Glusoafe2. Historical controls’ data will be retrospectively harvested from the patients’ computerized record. They will be recruited within 2 years before the trial start.
**Inclusion / exclusion criteria:**	**Inclusion criteria:**<br>All patients ≥ 18 years old admitted in adult ICU- Geneva with<br>• An expected length of stay ≥ 72h<br>• At least 1 blood glucose (BG) measurement ≥10 mmol/l or 2 BG measurement ≥ 8.5 mmol/l<br>• Informed Consent signed by the subject/ legal representative, except for patients in the historical control group<br><br>**Exclusion criteria:**<br>• Lack of legal consent or consent withdrawn, except for patients in the historical control group<br>• Pregnant or breast feeding<br>• Diabetic ketoacidosis or hyperosmolar state<br>• Oral feeding<br>• Fulminant hepatic failure<br>• Medically contraindicated to receive rapidly acting insulin by intravenous (iv) infusion or iv injection

**Measurements and procedures:**	Screening will be done every day (Monday to Friday). Eligible patients will be randomly allocated to either intervention or control group. Before inclusion, all patients will be treated according to the ICU- Geneva Protocols.<br>**Collected data:**<br><u>Baseline at study inclusion day</u><br>– Body mass, height and body mass index (BMI)<br>– Age, gender<br>– Date and time of admission in the ICU<br>– Primary diagnostic<br>– Known chronic illness (diabetes, acute kidney injury (AKI), pancreatitis, tumor, etc.).<br>– Medication influencing glycaemia (oral antidiabetics' insulin, steroids, prednisone, etc.).<br>– Renal and hepatic status<br>– Glycaemia and lactate<br>– Vitals<br>– Severity score (APACHE II, SAPS II) at ICU admission<br><u>Daily data from inclusion day (maximum follow up of 15 days)</u><br>*When possible – Following routines measurement.*<br>– Glucose measurements<br>– Other laboratory: lactate, CRP, sodium (Na), potassium (K), glycohemoglobin (HbA1C), urea, creatinine, glomerular filtration rate (GFR), protein, albumin, ASAT, ALAT, phosphatase alkaline, γ-glutamyltranspeptidase (GGT), bilirubin<br>– Any events (intubation, extubation, length of surgery, infusions, cardiac events, infections, requirement for red blood cell (RBC) transfusion, hemodynamic rate (HR), organ failure)<br>– Vitals (blood pressure, temperature, heart rate etc.)<br>– Antibiotics usage<br>– Vasopressor<br>– Steroids<br>– Propofol® for sedation<br>– Glucose perfusion<br>– Insulin therapy including boluses.<br>– Body weight, BMI, Ideal body weight (if needed), presence of oedema.<br>– Energy expenditure (IC or predictive formula)<br>– Energy and protein targets over the day<br>– Prescription and administration (ml of product, kcal and grams of protein).<br>– Daily water balance<br>– Diuresis<br>– Diarrhoea, constipation<br>– Nausea, Vomiting, gastric residual<br>– Physical therapy<br>– Medical Research Council Scale (MRC Scale)<br><u>Upon ICU leave</u><br>– Duration of ICU stay<br>– MRC Scale at ICU discharge<br>– Alive status at ICU discharge<br>– Duration of hospitalization<br>– Alive status at hospital discharge<br>– BMI at hospital discharge

**Intervention:**	**Intervention group**: number of patients: **71.**<br>Glucosafe 2 software is used for 15 days or until the patient is discharged from the ICU or starts eating (oral feeding). Glucosafe 2 integrates the following data: body weight (BW), age, gender, type of diabetes, renal replacement therapy, serial blood glucose measurements, insulin therapy and the nutrition support (including non-nutritional calories). Based on those data and according to the mathematical model it contains, Glucosafe 2 will calculate patients' insulin sensitivity and will recommend the dose of insulin and nutrition required for each ICU patient. For more information, see point 8.1.1.
**Control intervention (if applicable):**	**Control group:** number of patients: **71**.<br>Standard care according to department (ICU-HUG) protocol. BG measurement will be made every 2 hours for all patients admitted in the ICU. Depending on the stability of those measurements, BG control will be made in a close or extended way according to our internal protocol.<br>Early enteral nutrition will be started within 48 hours if the medical situation allows it. Nutrition will be given in a progressive way with the aim of covering the energy and protein needs at the end of day 3. For more information, see point 8.1.2.<br>**Historical control group:** number of patients: **71**.<br>Retrospective data, with standard care before the beginning of the pilot study. Historical controls data will be retrospectively collected from the patients' computerized record. They will be recruited within 2 years before the trial starts.
**Number of subjects with rationale:**	Based on data collected routinely at the ICU between January and March 2017, the time in target was 68% (SD 21%) for patients under the standard of care. Considering an improvement of 10% of time in target as clinically important, we would need 71 patients by arm (213 in total) to detect such an effect with a study power of 80% and an alpha error of 5% (two-sided).
**Duration of the investigation:**	12 months
**Investigation schedule:**	Month Year of First-Participant-In (planned): January 2023<br>Month Year of Last-Participant-Out (planned): December 2023
**Investigator(s):**	**Dr. Claudia Paula Heidegger**, MD, Deputy head physician (Sponsor and principal investigator)<br>**Mme Aude de Watteville**, BSc dietician (co-investigator)<br>**M. Nicolas Parel**, MSc dietician (co-investigator)<br>**M. Yosr Karker**, IT manager (co-investigator)<br>**Dr. Angèle Gayet-Ageron**, MD, Deputy head physician (co-investigator)<br>**Dr. Nils Siegenthaler,** MD, Deputy head physician (co-investigator)<br>**Dr. Hannah Wozniak**, MD, clinical head (co-investigator)<br>**Prof. Ulrike Pielmeier**, PhD, biomedical engineer (co-investigator)<br>**Prof. Andreassen** Steen, PhD, Dr. Tech (co-investigator)
**Investigational Site(s):**	Single-centre: Department of acute Medicine (DMA), Division of intensive care, Geneva University Hospital (HUG).

**Statistical considerations:**	Continuous variables will be presented according to the study group by their mean ± standard deviation (SD), median, interquartile range and range. Categorical variables will be presented according to the study group by their frequencies and relative proportions. The primary outcome (time in target) will be compared between the three arms using Student t test or Mann-Whitney nonparametric test, if distributions is non-normal. For secondary outcomes, continuous variables will be compared between the three study groups using either Student t test or Mann-Whitney nonparametric test; categorical variables will be compared using either Chi-2 test, or Fischer exact test, depending on application criteria. Count data (number of hyperglycemic episodes) will be compared using Poisson regression model or negative binomial regression, if over-dispersed.<br>Significance level is set for a p-value of 0.05. All analyses will be performed using STATA IC 15.0 software.
**Compliance statement:**	This investigation will be conducted in compliance with the CIP, the current version of the Declaration of Helsinki [1], ISO14155 [2], ICH-GCP [3] (as far as applicable) as well as all national legal and regulatory requirements.

ABBREVIATIONS

AAU	Aalborg University
ADE	Adverse Device Effect
ADO	Oral antidiabetic drugs
ADE	Adverse Device Effect
AE	Adverse Event
AKI	Acute kidney injury
ALAT	Alanine-Aminotransferase
APACHE II score	Acute Physiology and Chronic Health Evaluation II score
ASR	Annual safety report
AUC	Area under the curve
ASAT	Aspartate-Aminotransferase
BG	Blood glucose
BMI	Body mass index
BSc	Bachelor of Science
BW	Body weight
CA	Competent Authority (e.g. Swissmedic)
CDMS	Clinical Database Management System
CEC	Competent Ethics Committee
CIP	Clinical Investigation Plan
ClinO	Ordinance on Clinical Trials in Human Research
ClinO-MD	Ordinance on Clinical Trials with Medical Devices
CRF	Case Report Form
CRP	C-Reactive Protein
CRRT	Continuous renal replacement therapy
DD	Device deficiency
DMA	Department of acute medicine
DSI	Information Systems Department
Ecrf	Electronic case report form
EE	Energy Expenditure
ePDMS	Electronic Patient Data Management System
EN	Enteral nutrition
GCP	Good Clinical Practice
GFR	Glomerular Filtration Rate
GGT	Gamma-glutamyltransferase
GMDN	Global Medical Device Nomenclature
GR2/GR3	General risk 2/3
GS2	Glucosafe 2
HbA1C	Glycosylated haemoglobin
HD	Haemodynamic

HR	Heart rate
HRA	Swiss Federal Human Research Act
HBP	High blood pressure
HUG	Geneva University Hospitals
IC	Indirect Calorimetry
ICF	Informed consent form
ICH-GCP	International Conference on Harmonisation of technical requirements for registration of pharmaceuticals for human use – Guidelines for Good Clinical Practice
ICU	Intensive care unit
ID	Identity
IFU	Instruction for use
ISO	International Organisation for Standardisation
IT manager	Information Technology Manager
IV	Intravenous
K	Potassium
MD	Medical device
MDR	European Regulation on medical devices
Mg	Magnesium
MRC	Medical Research Council
MSc	Master of Science
Na	Sodium
PI	Principal investigator
PN	Parenteral nutrition
PO4	Phosphates
RBC	Red blood cell
RDBMS	Relational Database Management System
SAE	Serious adverse event
RMS	Root Mean Square
SADE	Serious Adverse Device Effect
SAPS II	Simplified Acute Physiology Score
SD	Standard deviation
SIRS	Systemic inflammatory response syndrome
SPN	Supplemental parenteral nutrition
SVD	Source Data Verification
UIC	Clinical investigation Unit
USADE	Unanticipated Serious Device Effect
WHO	World Health Organization

INVESTIGATION SCHEDULE

Study Periods	Screening and Patient information	Consent (ICF) + inclusion	Intervention	Follow-up
Visit	0	1	2-17 (max)	18-19
Time (hour, day, week)	-2 to -1 day	0	From inclusion day during max 15 days	ICU discharge and Hospital discharge
Inclusion and exclusion criteria	X			
Patient or legal representative Information	X			
Patient or legal representative consent		X		
Randomisation		X		
Demographics		X		
Anthropometrics		X	X	X
Details of hospitalisation		X		
Diagnosis		X		
Medication		X	X	
Laboratory Tests		X	X	
Vitals		X	X	
Severity score		X		
Glycaemia			X	
Events			X	
Energy and protein intake			X	
Elimination			X	
Digestive tolerance			X	
Physical therapy			X	X
(Serious) Adverse Events, Adverse device effects			X	X
Device Deficiencies			X	X

* The information reported during Visit 1 is the most recent information available at the time of this visit.

1. INVESTIGATION ADMINISTRATIVE STRUCTURE

1.1 Sponsor, Sponsor-Investigator

Claudia-Paula Heidegger, MD, Deputy head physician

Representative of the Geneva University Hospitals

Rue Gabrielle-Perret-Gentil 4, 1211 Geneva 14, Switzerland

Tel: +41-22 372 74 40

Fax: +41-22 382 74 70

Email: claudia-paula.heidegger@hcuge.ch

1.2 Principal Investigator(s)

Claudia-Paula Heidegger, MD, Deputy head physician

Department of Acute Medicine (DMA)

Division of intensive care, Geneva University Hospitals

Rue Gabrielle-Perret-Gentil 4, 1211 Geneva 14, Switzerland

Phone: +41-22 372 74 40

Email: claudia-paula.heidegger@hcuge.ch

1.3 Statistician ("Biostatistician")

Angèle Gayet-Ageron, MD, Deputy head physician

Unité d'Appui Méthodologique, Geneva University Hospitals

Rue Gabrielle-Perret-Gentil 4, 1211 Geneva, Switzerland

Phone : +41-22 372 90 27

Email: Angele.Gayet-Ageron@hcuge.ch

1.4 Laboratory

No laboratory analysis will be requested during this study. Data recorded will correspond to the usual currently laboratory analysis requested in the clinical practice of the adult ICU of HUG. Therefore, analyses will be carried out by the laboratory collaborating with HUG.

1.5 Monitoring institution

EURL H Villemagne represented by

M. Hervé Villemagne

Clinical study management and monitoring

357 Route de Vesegnin

01280 Prevessin

France

1.6 Data Safety Monitoring Committee

A data safety monitoring committee is not planned for this study. The purpose of the study is "safety and efficacy" of a new medical device. The study terminates in case of: ethical concerns; insufficient subject recruitment; if 5 patients present with two severe hypoglycaemias (≤2.2 mmol/l) in the intervention group; 10 device deficiencies which prevent the use of Glucosafe 2 (GS2) for 4 hours; alterations in accepted clinical practice that make the continuation of the investigation unwise; early evidence of benefit or harm of the experimental intervention or because root mean square (RMS) prediction error of GS2 is greater than 26% for predictions of BG 2 hours into the future. Device deficiencies will be handled according to point 5.1 described in the GS2-IB.

1.7 Any other relevant Committee, Person, Organisation, Institution

Not applicable.

2. ETHICAL AND REGULATORY ASPECTS

The final positive decision of the CEC and of the CA on the conduct of the investigation will be made and given in writing to the Sponsor before the investigation can start. Additional requirements set by the authorities must be implemented.

2.1 Registration of the investigation

This study is registered on the International Clinical Trial Registry website: www.clinicaltrial.gov. This registry is listed in the WHO international Clinical Trials Registry. ID: NCT03890432. The study will also be registered in the Swiss National Clinical Trials Portal (SNCTP).

2.2 Categorisation of the investigation

Category C2 (study involving a medical device which does not bear a conformity mark yet) (Art.6 ClinO-MD) [4].

2.3 Competent Ethics Committee (CEC)

The Sponsor-Investigator will submit the investigation to the CEC and obtain ethical committee approval before the start of the investigation. The PI ensures that approval from the CEC is obtained and filed in the Investigator site file before the investigation starts.

2.3.1 Reporting duties to the Competent Ethics Committee

Amendments are reported according to Art. 15 ClinO-MD [4] (See also 2.10).

The regular or premature end of the investigation as well as the interruption of the investigation is reported to the CEC within 15 days (within 24 hours if it is due to security reasons) (Art. 36 ClinO-MD). The reasons for a premature end or an interruption have to be explained.

A final report shall be submitted within one year after the regular end of the investigation and within 3 months after a premature end of the investigation (Art. 37 ClinO-MD) [4].

2.4 Competent Authorities (CA)

The Sponsor-Investigator will submit the investigation to the CA and obtain regulatory approval before the start of the investigation. The PI ensures that approval from the CA is obtained and filed in the Investigator site file before the investigation starts.

2.4.1 Reporting duties to the competent authorities

Amendments are reported according to Art. 20 ClinO-MD [4] (see also 2.10).

The regular or premature end of the investigation as well as the interruption of the investigation is reported to the CEC within 15 days (within 24 hours if it is due to security reasons) (Art. 36 ClinO-MD) [4]. The reasons for a premature end or an interruption have to be explained. Refer to chapter 10 for safety reporting.

2.5 Ethical Conduct of the Investigation

The investigation will be carried out according to the CIP and with principles enunciated in the current version of the Declaration of Helsinki [1], the European Regulation on medical devices 2017/745 (MDR) [5], the Norms ISO14155 [2] and ISO14971 [6], the ICH-guidelines of Good Clinical Practice (GCP) [3] as applicable, the Swiss Human Research Act (HRA) [7] and its Ordinances and Swiss regulatory authority's requirements [4, 8]. The CEC and the CA will receive the Annual Safety Report (ASR) and interim reports and be notified about investigation stop/end in agreement with local requirements.

2.6 Declaration of interests

The sponsor and the HUG clinical investigators have no conflict of interest to declare in relation to this

clinical trial. The manufacturer, Aalborg University, is a public academic institution and does not receive financial support from private sources of any kind in support of this clinical trial. All rights to the device remain with Aalborg University for the duration of the trial. Public funding from a governmental fund has been received in January 2021, and the contract with the funding organization requires Aalborg University to investigate ways to commercialize the device in the future. On that basis it is declared that the manufacturer, Aalborg University, has a commercial interest in this clinical trial.

2.7 Patient Information and Informed Consent

The investigators explain to each subject the nature of the investigation, its purpose, the procedures involved, the expected duration, the potential risks and benefits and any discomfort it may entail. Each subject and/or legal representative is informed that the participation in the investigation is voluntary and that he/she may withdraw from the investigation at any time and that withdrawal of consent will not affect his/her subsequent medical assistance and treatment. The subjects and/or legal representatives are informed that he/she can ask any question, and consult with family members, friends, their treating physicians or other experts before deciding about their participation in the investigation. A deadline of 24 hours will be given to the legal representative or to the independent physician after presentation of the study to give or not their consent. For patients, once they are capable of consenting, a 48-hour delay after presentation of the study will be given.

The subjects/ legal representatives are informed that authorised individuals other than their treating physician may examine his/her medical records.

All subjects and/or legal representative are given a subject information sheet and a consent form describing the investigation and providing sufficient information for the subjects to make an informed decision about their participation in the investigation.

The formal consent of a subject/ legal representative or doctor not associated with the study, using the approved consent form, is obtained before the subject is submitted to any investigation procedure.

The subject/ legal representative should read, understand, and voluntarily agree before signing and dating the informed consent form, and is given a copy of the signed document. The consent form is signed and dated by the subject and the PI (or her/his designee). The signed consent form is retained as part of the investigation records.

As this study involves patients in an emergency situation, most patients will be unable to give their informed consent at the time of inclusion. Nevertheless, participants should provide their own consent as soon as they regain their capacity to consent, in order to confirm their agreement for the continuation of their participation in the study.

The consents' procedure for participants to intervention or control group will be carried out as follows:

1) It will be ensured that the patient did not express his/her right to refuse to participate in research projects in any identifiable manner (electronic patient's record or according to relatives). This information will be clearly documented in the patient's medical files.
2) If the patient has his capacity to discern, he/she will give his/her own consent before inclusion in the study.
3) If the patient is unable to consent at the inclusion time, the consent will be obtained from their legal representatives in a proxy consent. Waiting for the legal representative consent, a doctor not associated with the study is called to safeguard the interests of the person concerned (art 30 HRA [7]. The enrolment of the patient in the study can be made when the independent physician authorisation is obtained. This authorisation form must be signed by the independent physician and an investigator of the study and will be retained as part of the study records.
4) As soon as the patient has recovered his/her capacity to consent, he/she will be asked to sign a post-hoc consent form, which will be retained as part of the study records.

2.7.1 Request for authorization under Article 34 of the HRA

For the participants in the retrospective control group, we ask for permission to use the data without the patient's consent in accordance with Article 34 of the HRA [7]. In fact, we want to analyse 71 data files from patients who were admitted to the ICU within 2 years before the start of the study. The process of obtaining post-hoc consent would be extremely complex as ICU mortality can be as high as 20%. The exclusion of patients for whom we cannot obtain consent would lead to a selection bias.

The project sponsor/investigator confirms that no patient health-related data will be utilized in this study from patients that refused participation (in written or documented oral refusal) in research studies.

Important information regarding informed consent and the use of collected data:

- If the participant or representative refuses post-hoc or proxy consent, the participant will be withdrawn from the study. The data collected so far will be destroyed and will not be analysed (ClinO Art. 17, alinea 3) [8]. If a participant or representative withdraws his/her consent, the same process will apply.
- If no representative is identified and no proxy consent could be obtained, the patient will be withdrawn from the study and his/her data will be destroyed and will not be analysed. If the patient recovers his/her capacity to consent and declines to give consent, he or she will be withdrawn from the study and the procedure outlined above will apply.
- If a participant already enrolled in the study dies before consent or refusal could be obtained from him/ her or a representative, the personal health information collected may be used only if the participant has consented to the use of his/her personal health data for research purposes in an advance directive or other form.

2.8 Subject privacy and confidentiality

The investigators affirm and upholds the principle of the participant's right to privacy and that they shall comply with applicable privacy laws. Especially, pseudo anonymity of the participants shall be guaranteed when presenting the data at scientific meetings or publishing them in scientific journals.

Individual subject medical information obtained as a result of this study is considered confidential and disclosure to third parties is prohibited. Subject confidentiality will be further ensured by utilising subject identification code numbers to correspond to treatment data in the computer files.

For data verification purposes, authorised representatives of the Sponsor, the CA or a CEC may require direct access to parts of the medical records relevant to the investigation, including subjects' medical history.

2.9 Early termination of the investigation

The Sponsor may terminate the investigation prematurely according to certain circumstances, for example:

- Ethical concerns
- Insufficient subject recruitments
- More than 5 patients with two episodes of severe hypoglycaemia (≤2.2 mmol/l) in the intervention group
- 10 device deficiencies that prevent the use of GS2 for 4 hours
- Changes in accepted clinical practice that are too large and make it unwise to continue the investigation
- Early evidence of benefit or harm of the experimental intervention
- Root mean square (RMS) prediction error of GS2 is greater than 26% for predictions of BG 2 hours into the future (see point 3.4 for rationale)

2.10 Clinical investigation plan amendments

Substantial amendments are only implemented after approval by the CEC (Art. 15 ClinO-MD) [4] and after approval by the CA also (Art. 20 ClinO-MD) [4]. The use of waivers from the CIP is prohibited (Annex XV, Chapter 2, Art. 3.10 MDR).

Under emergency circumstances, deviations from the CIP to protect the rights, safety and well-being of the subjects may proceed without prior approval by the Sponsor, the CEC and the CA. Such deviations shall be documented and reported to the Sponsor, the CEC and to the CA within 2 days (Art. 34 ClinO-MD) [4].

All non-substantial amendments are communicated to the CEC together with the Annual Safety Report (ASR) (Art. 15 ClinO-MD) [4], and for category C clinical investigations to the CA as soon as possible (Art. 20 ClinO-MD) [4]. The ASR shall include any deviations from the CIP that may have affected the rights, safety or well-being of the subject or the scientific integrity of the investigation (ISO14155) [2].

2.11 Deviation from the Clinical Investigation Plan

The use of waivers from the CIP is prohibited. Investigator is not allowed to deviate from the CIP, except

as specified in point 2.10. All deviations occurring will be followed in the deviation log.

3. BACKGROUND AND RATIONALE

3.1 Background and Rationale for the clinical investigation

The survival and the outcomes of critically ill patients are strongly influenced by the management of insulin therapy and the nutritional support. All improvements in these care processes and specific therapeutic interventions result in a decreased ICU morbidity and mortality rate [9-11]. Hypo- or hyperglycaemia are related to higher mortality, especially in non-diabetic patients [12, 13]. An association between high blood glucose (BG) variability and increased infection rates and mortality were also observed [14, 15]. In addition, inadequate nutritional support may also contribute to protein loss and muscle wasting, which may lead to post-ICU weakness [16-18] and to a further increase of the ICU and post-ICU morbi-mortality [19-21]. These ICU neuromuscular complications worsen the general clinical outcomes, and are linked to an increased mortality, persistent functional limitations, and cognitive impairment. The reported incidence varies between 25 and 100% [6].

International professional societies have published evidence-based guidelines for BG control and nutritional support for ICU patients [22-24], but their implementation and the adherence to local guidelines remains very challenging. Implementing BG control to local guidelines showed a low 50% adherence [25, 26] and despite nutritional recommendations, patients staying more than 72 hours in the ICU observed in a multicenter study received on average less than 60% of their estimated energy expenditure [27].

The Glucosafe 2 (GS2) software is a decision support system intended to help caregivers in achieving optimised BG control coupled to nutritional support. It is based on a mathematical model of the glucose-insulin metabolism. This model integrates the following data: body mass (BM), age, gender, type of diabetes, renal replacement therapy, serial blood glucose measurements, insulin therapy and the nutrition support (including non-nutritional calories). Based on these data, GS2 can calculate the patient’s insulin sensitivity and recommends the dose of insulin and nutrition required for each ICU patient.

This model should allow a meticulous glycaemia control and a diminution of glycaemia variability as well as the reduction of deleterious hyper- or hypoglycaemia events. Furthermore, the GS2 model allows to a certain degree to compensate cumulated energy and protein deficits after prolonged acute illness with high catabolism. In addition, GS2 provides information to supplement nutritional proteins by complementary amino acid perfusions in order to reach the protein target.

This pilot study aims to evaluate if the use of the GS2 software can increase the quality of the glucose control (“time-in-target”, stability of blood glucose, decrease of hyper- hypoglycaemia events) as well as the quality of nutrition therapy (reaching of energy and protein targets, decrease of energy and protein debts) in comparison with the actual local protocols.

3.2 Identification and description of the Investigational Medical Device

The name of the investigational product is GS2. The GS2 software is a class IIb decision support system. The version used in the protocol is release 1.0, GMDN code: 61087. The manufacturer is Aalborg University, Aalborg, Denmark. GS2 is not commercially available and is not CE-marked.

GS2 software is a medical device, which for the purpose of the clinical investigation will be installed at the bed-side computer running Centricity, which is the electronic Patient Data Management System (ePDMS) used in the ICU-Geneva. GS2 is not in physical contact with the patient, nor is it connected with other devices that are in physical contact with the patient (e.g. infusion pumps).

The GS2 system is designed to provide:

1) On-demand open-loop advice for personalized insulin therapy to regulate blood glucose in adult ICU patients based on predictions of the patient's BG using a physiological model of glucose and insulin pharmacodynamics, gastrointestinal absorption of nutrients and insulin release.
2) An overview of the glycaemic and nutritional history and current status of adult ICU patients through information automatically retrieved from the ePDMS and the GS2 database.
3) On-demand open-loop advice for setting of personalized nutrition targets and for achieving the

caloric and protein targets.

4) Balancing of insulin dosing and nutritional targets against each other.

5) Supporting the clinical workflow by maintaining a “To Do” list of upcoming tasks.

Indication: GS2 is intended for use in adult ICU patients fasting or on enteral and/or parenteral nutrition. In patients with neurological trauma, GS2 will increase the threshold for hypoglycaemia to safeguard the patients against cerebral hypoglycaemia.

Contraindications: GS2 is not intended for patients on oral feeding or pregnant patients.

Detailed criteria for inclusion/exclusion are given in sect. 7.1.

The device is intended for use by physicians and nursing staff trained in the use of GS2. The caregiver can accept or reject the advice ("open-loop advice"), mitigating issues of safety and effectiveness.

GS2 is not intended to monitor the performance of the insulin and nutrition pumps.

3.3 Preclinical Evidence for GS2

Glucosafe, the predecessor of GS2, was a clinical research tool with focus on improving blood glucose control, in particular during the acute phase of the ICU stay, where insulin for blood glucose control is most needed. GS2 is intended for use as a clinical medical device, applicable for most ICU patients both during the acute phase and the recovery phase, where the focus on adequate nutrition is increased. This has necessitated modifications to turn Glucosafe into GS2. The modifications are listed and discussed in detail in GS2's investigator's brochure (GS2-IB).

Both preclinical and clinical testing of Glucosafe was made in the past. Due to the modifications, equivalence is not claimed between Glucosafe and GS2, but the closeness of Glucosafe's model to GS2's model and the fact that the prediction error of Glucosafe has been studied to some extent and in different cohorts (explained in Section 3.3.1), motivates us to consider evidence for Glucosafe as preclinical evidence for GS2. Moreover, Glucosafe's BG prediction error curve is used as a factor for early termination of the clinical trial should GS2 fail to reach the expected prediction accuracy (as explained in Section 2.9). We summarize the available preclinical and clinical testing in Sections 3.3.1 and 3.3.3.

3.3.1 Preclinical testing of Glucosafe

A review of the preclinical testing of Glucosafe has shown:

a) Glucosafe's physiological model can simulate the blood glucose data during normoglycaemia and during hyperglycaemia [28]. This ability is due to the saturation of insulin action incorporated in Glucosafe's physiological model [28, 29]. This gives a rationale for believing that Glucosafe's model may also be superior for simulating BG during changes in BG and insulin concentration.

b) Two retrospective analyses [30, 31] tested the ability of Glucosafe's physiological model to predict BG concentration of critically ill patients into the future and quantified the accuracy of these predictions in different patient cohorts and for different prediction time lengths. We found that the prediction error becomes larger the longer blood glucose is predicted into the future, as seen from the graphs in Figure 1 both on the left and right panel. We also found that there may be different prediction errors for different cohorts, as seen on the right panel ofFigure 1. FromFigure 1, we quantified the prediction error of Glucosafe's physiological model to be in the range of 10% to 17% for predictions of 1 hour into the future, and 17% to 26% for predictions of 2 hours into the future.

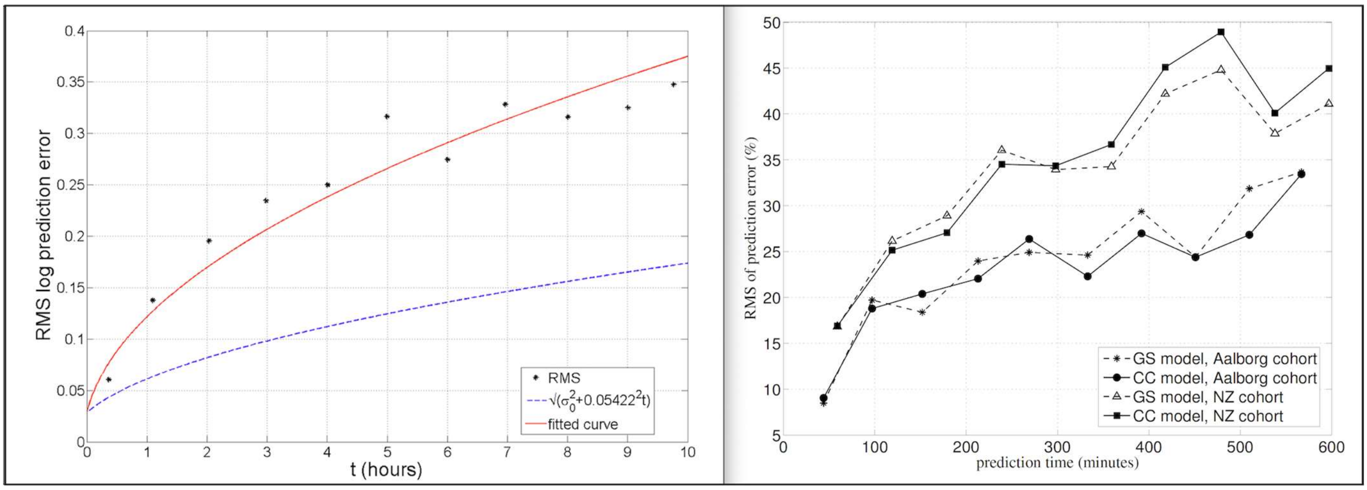

Figure 1 Analysis of prediction error from retrospectively predicting BG with Glucosafe's physiological model. Left: The root mean square (RMS) of the natural logarithm of the BG prediction error is plotted against prediction time in hours on the x-axis [31]. Data are from 10 randomly selected trauma and/or neurologic ICU patients. Right: The RMS of the relative prediction error is plotted against prediction time, for two different physiological models and patient cohorts. The Glucosafe model is denoted "GS model" [30].

3.3.2 Conclusion from the preclinical evidence for Glucosafe

Glucosafe's physiological model can simulate BG in different cohorts under normoglycaemic, hyperglycaemic, and hypoglycaemic conditions. Glucosafe predicts BG with good accuracy, but the prediction error depends on the prediction time. In different cohort, prediction errors ranged from 10% to 17% for predictions of 1 hour, 17%-26% for predictions of 2 hours, and 21%-29% for predictions of 3 hours.

3.3.3 Clinical evidence for Glucosafe

Glucosafe has been clinically studied in three prospective pilot studies. Two were published [32, 33] and the third study is described in the following abstract [34]. Although the studies are clinical, we consider them pre-clinical in relation to GS2, since the addition of advice on protein intake in GS2 makes Glucosafe and GS2 non-equivalent. The studies were conducted between 2009 and 2011 with ethics approval from the local ethics boards.

c) In the first of these studies, conducted in 2009 [32], 10 consecutively recruited hyperglycaemic patients (2 BG > 8 mmol/l or 1 BG > 10 mmol/l) from a neuro-trauma ICU were treated according to Glucosafe advice for 12-14 hours. Patients had an average APACHEII score of 12 (range 3-18), most with subarachnoid or intracranial haemorrhage. Hypoglycaemia (BG < 3.5 mmol/l) was not observed. Both mean BG and BG variability during the intervention period was lower (7.0 ± 1.1 mmol/l) compared to the 24h-preintervention period (8.6 ± 2.4 mmol/l; p < .01) and also compared to the 24h-postintervention period (7.4 ± 1.5 mmol/l; p = .03) (Figure 2). Nine of ten patients reached the normal BG target range (defined as 4.4-6.1 mmol/l in this study) after a mean of 5 hours with Glucosafe advice. The caregivers accepted 73% of Glucosafe advice unaltered, and the remaining were partially accepted.

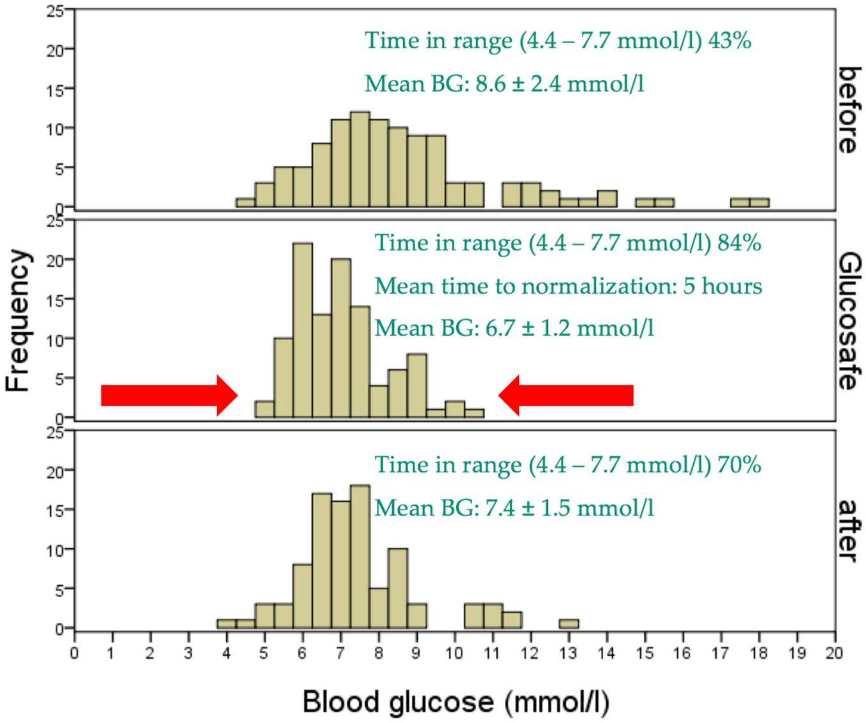

Figure 2. Distribution of BG during 24h-preintervention (before), 12-14h-intervention (Glucosafe) and 24h-postintervention (after).

d) The second study [33] was planned as a randomized controlled trial of 40 consecutively enrolled patients in a neuro-trauma ICU, but was discontinued because organizational challenges at the clinical partner site made the successful completion of the trial unlikely. Data from 12 patients (6 intervention, 6 control group patients) were analysed, all with traumatic brain injury, polytrauma, or subarachnoid haemorrhage. Patients had an average SAPSII score of 39 (range 21-60).

Data were collected for four days. The BG target range 5-8 mmol/l. Nutritional targets were individually prescribed by the attending physician and were between 25-30 kcal/d per kg ideal body weight. The mean BG was significantly lower (7.0 ± 1.2 mmol/l) in Glucosafe patients than in control patients (8.0 ± 1.2 mmol/l). No adverse events including hypoglycaemia were reported in either group. In the intervention group, 170 (76%) measured BG values were in the 5-8 mmol/l target band, with the lowest BG value at 4.2 mmol/l. In the control group, 171 (51%) measured BG values were in the 5-8 mmol/l target band and the lowest BG was 4.0 mmol/l. The Glucosafe group was closer to isocaloric feeding (93.5 ± 15% of EE) than the control group (129.5 ± 29.4% of EE), but the difference was not significant.

e) In the third pilot study [34], Glucosafe's advice for insulin infusion was administered to 13 hyperglycaemic surgical and medical ICU patients and glycaemia was compared to the pre- and post-intervention days. The BG target range was 5-8.3 mmol/l. Nutrition was provided according to the department guidelines. Fig. 3 shows the cumulated BG measurements over the data collection periods.

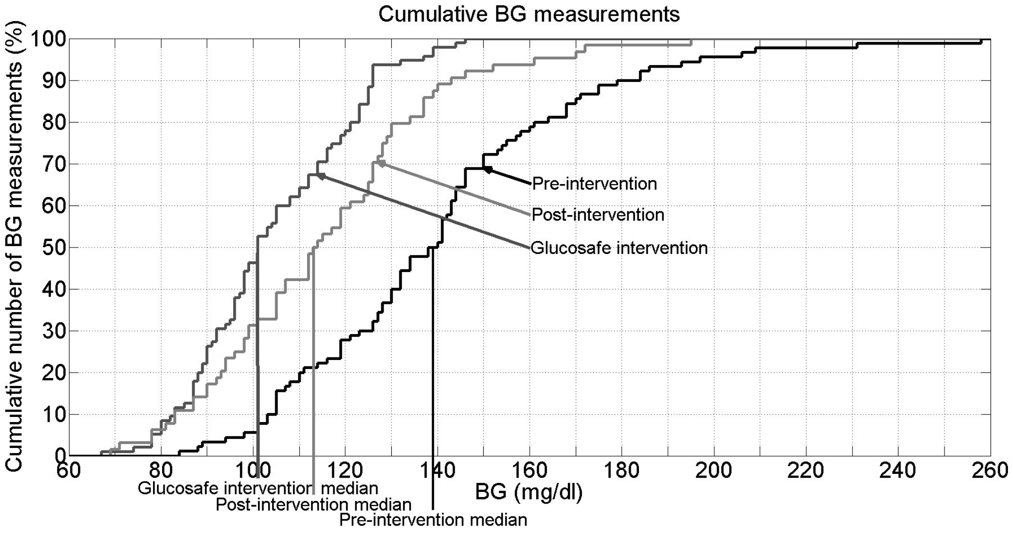

Figure 3. Distribution of BG during 24h-preintervention (before), 12-14h-intervention (Glucosafe) and 24h-postintervention (after).

During Glucosafe intervention the mean and SD of BG (5.8 ± 0.9 mmol/l; N=99) was lower than during pre-intervention (7.8 ± 1.8 mmol/l; N=86) and during post-intervention (6.4 ± 1.4 mmol/l; N=56). No hypoglycaemic events occurred.

3.3.4 Summary of the clinical evidence for Glucosafe

The studies mentioned in Section 3.3.3 above showed that advice by Glucosafe lowered the mean BG and increased the time in the BG target range. In the three studies the BG target ranges were 4.4-7.7 mmol/l, 5-8 mmol/l and 5-8.3 mmol/, respectively.

In study d, where Glucosafe was used to advice both on insulin and caloric intake, the advice from Glucosafe brought the caloric intake closer to the recommended isocaloric intake, where the caloric intake matches the estimated energy expenditure.

3.4 Clinical Evidence to Date for GS2

In the GS2-Clinical Evaluation Report, no clinical testing of GS2 or any equivalent medical device has been identified. Therefore, no claims are currently made for GS2. The performance and safety of GS2 will be assessed through a clinical trial for which a pre-market approval has been applied for at the relevant competent authority.

As mentioned in point 3.3 "Preclinical evidence for GS2", all preclinical tests were performed with Glucosafe, the predecessor of GS2. The predictive accuracy of GS2 will be confirmed by analysis of the historical control group before the prospective phase of the study is initiated.

GS2's predictions of the BG concentrations may be less accurate than assumed. The BG prediction error of GS2 is assumed to match to the error of the GS2 predecessor, Glucosafe (see Section 3.3). The assumed error curve is shown in Fig. 4 below, which was determined by several small retrospective studies [30, 31] of model-based predictions of BG in different ICU patient cohorts (not including ICU-Geneva patients). As with its predecessor Glucosafe, GS2 uses prediction error to calculate the maximum time until the next blood glucose measurement to keep the risk of hypoglycemia below the acceptable maximum risk level (see Section 8.1.1 for details).Thus, a larger prediction error shortens the time to the next measurement, which could ultimately increase the frequency of BG measurements to an unacceptable and undesirable level. From Fig. 4 it can be read that the RMS prediction error of Glucosafe was about 26% for predictions of BG 2 hours into the future. The RMS prediction error will be estimated from the historical control group and a new curve will be drawn to be compared with the curve in Fig. 4. It is expected that GS2 performs equally well or better than its predecessor. If the RMS prediction error of GS2 is greater for the historical control group, the sponsor will decide to discontinue the GS2 study. The cut-off point set to 2 hours is based on the frequency of BG measurements recommended by ICU-Geneva's glycaemic control policies (see Sect.8.1.2).

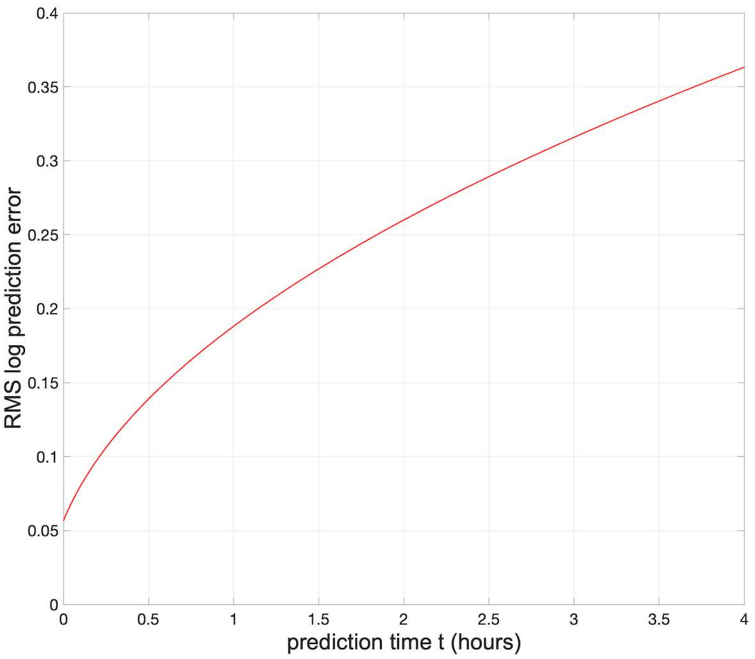

Figure 4 Maximum acceptable RMS prediction error curve for predictions of BG in the historical control group. The curve is derived from pre-clinical evidence with GS2's predecessor, Glucosafe. For predictions 2 hours into the future, the curve allows the RMS prediction error to be at a maximum of 26%.

3.5 Justification for the design of the clinical investigation

Reduction of hyper- and hypoglycaemia reduces mortality and adequate nutritional support reduces post-ICU weakness as well as ICU and post-ICU morbi-mortality, as discussed in sect. 3.1, The intended purpose of the study is therefore to assess whether GS2 safely and effectively can reduce hyper- and hypoglycaemia and can bring the achieved nutritional support closer to the nutritional guidelines. The achievement of these study objectives (sect. 4) will be evaluated through the calculation of a number of study outcomes (sect. 5), which will be used to make comparisons between the patients in:

1) GS2 intervention group
2) Control group
3) Historical control group (sect. 3.6)

3.6 Explanation for choice of comparator

In the GS2 intervention group the clinicians are advised by GS2 and in the control group the patients are treated in accordance with the department's guidelines. There may be a cross-over effect from the GS2 advice, which potentially may influence the clinicians' treatment of the patients in the control group. To be able to assess this cross-over effect a historical control group is included in the trial.

3.7 Risk evaluation (Risk-to-Benefits rationale)

A device risk analysis and risk assessment has been conducted according to EN ISO 14971. Risks were identified by class: General risks, software risks, usability risks and installation risks according to EN 14971, EN62304, EN62366-1 and EN14971, respectively. All identified risks related to the four events: Hypoglycaemia, hyperglycaemia, overfeeding or underfeeding. After mitigation of risks, all risks were minor, except two general risks, GR2 and GR3, both related to hypoglycaemia. These two risks therefore require justification.

The justification of **GR2**, as stated in the GS2-Risk Management Report, showed that the expected risk of a patient experiencing hypoglycaemia is 2%, which is smaller than the 5% reported in IIT trials in the control group and much smaller than what is reported for the intervention group. The risk of 2% depends on the setting Hypoglycaemia Risk Maximum (GS2- SDS03-Settings in Appendix 7.2). The Hypoglycaemia Risk Maximum is set to 0.05%. This is the risk that a given BG measurement, made at the due time calculated by GS2, is hypoglycaemic, as described in GS2-SDS06-BG prediction error and BG due time. Assuming an average of 40 BG measurements per patient, then the patients risk of hypoglycaemia can be estimated as 0,05% * 40 = 2%.

The justification of GR3, as stated in the GS2-Risk Management Report, showed that the occasional hazard of not recognizing a type 1 diabetes patient is outweighed for that patient by the expected medical benefit of using GS2.

The final Risk Management Report in the GS2-Risk Management File state that the GS2 software has a Class B safety classification and that after all possible efforts to mitigate risks only two unacceptable risks remained, both general risks and both justifiable.

Moreover, as GS2 only gives advice and cannot control directly the insulin management, the risk remains lower than with an autonomous software.

3.8 Justification of the choice of the investigation population

The Glucosafe 2 software has been developed to meet the specific needs of critically ill ICU patients. The pre-clinical and clinical studies performed with its predecessor, Glucosafe, also have been made in this population to evaluate its predictive accuracy. The population of critically ill patients is characterized by a severe catabolic state due to a generalized inflammation, with high insulin resistance and increased proteolysis. In the acute phase, the patients' endogenous reserves (glucose, amino acids and fatty acids) are massively mobilized, and not taking account of this process may lead to overfeeding during this acute phase. Glucosafe 2 takes into account the entire metabolism of intensive care patients in every phase and accordingly provides advice on both blood glucose management and nutritional therapy [9, 13]. All of these are reasons why Glucosafe 2 should be tested specifically in intensive care patients and cannot be extended to other "general patient populations" that do not have these prerequisites.

4. CLINICAL INVESTIGATION OBJECTIVES

4.1 Overall Objective

The purpose of this study is to evaluate the performance and the security of GS2 software in comparison to the actual clinical practice (according to local protocols). The aim is to improve nutrition management and BG control during the ICU stay.

4.2 Primary Objective

The primary objective of the experimental study is to assess whether GS2 improves the blood glucose control and monitoring, which is reflected in an increased time on target (glycaemia between 5.0 and 8.5 mmol/l) compared to standard care.

4.3 Secondary Objectives

The secondary objectives are to assess whether GS2 stabilizes BG and reduces the variability in BG measurements and improves nutritional management by increasing the achievement of the daily energy and protein target by limiting the cumulated energy and protein debt compared to the standard of care.

4.4 Safety Objectives

The study will assess the safety of GS2 by decreasing the hypoglycaemic and hyperglycaemic episodes in comparison to the standard of care.

5. CLINICAL INVESTIGATION OUTCOMES

5.1 Primary Outcome

Time-in-target (range: 5.0 to 8.5 mmol/l) per patient and in the cohort.

For the purpose of calculating time-in target, BG values are considered constant from one measurement to the next. The time-in-target is defined as the cumulative time in the 5 to 8.5 mmol/l range divided by the time from the first BG measurement to 2 hours after the last BG measurement.

5.2 Secondary Outcomes

Glycaemic “variability”:

- Mean and SD of BG measurements per day and per patient during the enrolment period
- Maximum of daily BG difference

Achievement of nutritional targets.

- Estimates of energy expenditure, protein requirements, caloric target and protein target are made on a daily basis in the intervention and control groups, as detailed in sect. 8.1.1 and 8.1.2, respectively.
- The caloric target for the day is reached when of 80-100% of the target is achieved.
- The protein target for the day is reached when of 80-100% of the target is achieved.
- Cumulated energy debt, defined as daily energy expenditure minus caloric intake, cumulated over the enrolment period.
- Cumulated protein debt, defined as daily protein requirement minus protein intake, cumulated over the enrolment period.

Prediction of blood glucose (BG)

- Root mean squared prediction error as a measure of the differences between BG predicted by GS2’s metabolic model and measured BG. The prediction error will be calculated, as a function of time elapsed since last BG measurement, per patient, per cohort (sect. 9.2.3).

5.3 Other Outcomes of Interest

Workload:

- Frequency of daily and cumulated BG measurements per patient and in the cohort during the patient’s enrolment period (duration of the ICU stay with a max of 15 days).
- Frequency of daily and cumulated adjustments of insulin and nutrition pump settings during the patient’s enrolment period.

Compliance (only in intervention arm)

- Number of episodes per patient and in the cohort where a BG measurement is not followed up within 30 minutes by an accepted GS2 advice (during the enrolment period)
- Number of episodes per patient and in the cohort where GS2 advice was accepted by the nurse but pumps were not set according to the advice within 30 min by the nurse (nurse did not notice, was too busy, forgot, or misinterpreted advice = true errors)
- Number of advices given by GS2 which were accepted, accepted with modification or rejected

5.4 Safety Outcomes

Hypoglycaemia

- Overall number and percentage of mild (≤3.2 mmol/l), and severe (≤2.2 mmol/l) hypoglycaemic events per patient and in the study.
- Number and percentage of mild (≤3.2 mmol/l), and severe (≤2.2 mmol/l) hypoglycaemic events due to non-compliance (only in intervention arm)

Hyperglycaemia (>8.5 mmol/l)

- The time taken to normalize blood glucose will be the time from enrolment in the study to the time of the first of two consecutive BG measurements ≤8.5 mmol/l

- Percentage of time in the ICU with hyperglycaemia (BG > 8.5 mmol/l) before and after normalization per patient and in the cohort.
- Percentage of time in the ICU with hyperglycaemia (BG > 8.5 mmol/l) before and after normalization per patient and in the cohort due to non-compliance (only in intervention arm)
- Number of hyperglycaemic episodes after normalization per patient and in the cohort
- Number of hyperglycaemic episodes after normalization per patient and in the cohort due to non-compliance (only in intervention arm)

6. CLINICAL INVESTIGATION DESIGN

6.1 General clinical investigation design and justification of design

This study is an unblinded randomized controlled trial that aims to evaluate the performance and the safety of a new bedside tool: GS2 in comparison to the standard of care.

Data from the historical control group will be analysed retrospectively for 71 patients hospitalized in the ICU at the same period within 2 years before the study. The historical control group is included for two reasons. The first is to be able to assess cross-over effects. These may happen in the randomized control group, if the caregiver's behaviour in this group is influenced by the caregiver's exposure to the GS2 system, when the caregiver is using the system in the intervention group.

The second reason is to confirm the predictive accuracy of GS2. As described in section 3.3.1 there are modest differences between the prediction errors estimated from the three patient cohorts previously analysed with Glucosafe. It is therefore appropriate to confirm that the prediction error of GS2 is similar or smaller compared to Glucosafe's. In case the prediction error is smaller, it will be possible to reduce the frequency of blood glucose measurements, as described in IB 2.5 "Mechanisms of action". The frequency of blood glucose measurements is one of the outcomes for workload (section 9.2.3). This reduces workload and the amount of blood drawn from the patient. In case the prediction error is larger, it is a reason for discontinuation or modifications of the intervention, as noted in Section 2.9.

The analysis of data from the historical control group will start as soon as the authorization of the study has been obtained. Enrolment for the intervention and control groups will start when the retrospective analysis of the historical control group has been completed. Enrolment is expected to take 8 months. The data and the results will then be analysed and submitted for publication.

6.2 Methods for minimising bias

6.2.1 Randomisation

Randomization (1:1) will be stratified by the gravity score APACHE II (mild/moderate/severe) and by the diabetic status (diabetes/non diabetic patients). A randomization list using block randomization with variable blocks size 2, 4, or 6 will be computer-generated by an independent statistician.

6.2.2 Blinding procedures

Not applicable.

6.2.3 Other methods for minimising bias

The study includes a historical control group to have an independent way of assessing possible "cross-over" effects from the intervention group to the control group. Historical controls' data will be retrospectively harvested from the patients' computerized record. They will be recruited within 2 years before the trial start.

6.3 Unblinding Procedures (Code break)

Not applicable.

7. CLINICAL INVESTIGATION POPULATION

7.1 Eligibility criteria

Subjects fulfilling all the following <u>inclusion</u> criteria are eligible for the investigation:
All patients ≥ 18 years old admitted in adult ICU- Geneva with

- An expected length of stay ≥ 72h
- At least 1 blood glucose (BG) measurement ≥10 mmol/l or 2 BG measurement ≥ 8.5 mmol/l
- Informed Consent signed by the subject/ legal representative, except for patients in the historical control group

The presence of any one of the following <u>exclusion</u> criteria will lead to the exclusion of the subject:

- Lack of legal consent or consent withdrawn, except for patients in the historical control group
- Pregnant or breast feeding*
- Diabetic ketoacidosis or hyperosmolar state
- Oral feeding
- Fulminant hepatic failure
- Medically contraindicated to receive rapidly acting insulin by intravenous (iv) infusion or iv injection
  *A blood pregnancy test (blood beta-hCG) will be performed in all women of childbearing potential before entering the study.

7.2 Recruitment and screening

7.2.1 Intervention and control groups

Screening for inclusion into the intervention and the control groups will be done once a day (Monday to Friday) expect for the run-in phase. If more patients respect the eligibility criteria, priority will be given to people whose relatives can be reached at first. Eligible and consent patients will be randomized to either intervention group or control group. A deadline of 24 hours will be given to the legal representative or to the independent physician after presentation of the study to give or not their consent. For patients, once they are capable of consenting, a 48-hour delay after presentation of the study will be given. Before inclusion, all patients are treated according to the ICU- Geneva Protocols.

We will perform a “run-in phase” for the first 3 patients randomized in the intervention group. For these patients, we will ensure that the user asks for one advice in GS2 after each blood glucose measurement. Daily, one of the co-investigators will extract the GS2 data and check that all the advice given concerning insulin and glucose are correct according to GS2 predefined settings and if these are clinically acceptable. After the run-in period, if the GS2 recommendations are considered clinically acceptable, the control will be spaced at once a week. In the event of problems noted, the daily control will be maintained.

7.2.2 Historical control group

The patients in the historical control group will be recruited retrospectively as consecutive eligible patients, beginning within 2 years before the start of the recruitment into the intervention and the control groups.

7.3 Assignment to investigation groups

Once eligibility criteria have been verified and informed consent signed, patients will be randomized in one of the two study arms using randomization program in the secuTrial® platform.

7.4 Criteria for withdrawal / discontinuation of subjects

Once started in either intervention or control arm, the study protocol will continue until one of the following circumstances is encountered:

1- Death; or
2- Withdrawal of life support; or
3- Consent withdrawal

8. CLINICAL INVESTIGATION INTERVENTION

8.1 Identity of the medical device under investigation

8.1.1 Experimental Intervention (medical device)

The name of the investigational product is GS2. The version used in the protocol is release 1.0 GMDN code: 61087. The name of the manufacturer is Aalborg University, Aalborg, Denmark. GS2 software is a medical device, which during the clinical trial will be installed at the bed-side computer running Centricity, which is the electronic Patient Data Management System (ePDMS) used in the ICU-Geneva. The function of GS2 is affected by the settings of GS2. The settings supplied by the manufacturer are given in Appendix 5, which contains a copy of the document GS2-Settings, which is a part of the Technical file for GS2. The GS2-Site-Acceptence-Test-Plan requires a review by the Sponsor to ensure that the settings used in the protocol are adapted to the workflow and to the requirements of the protocol. Modifications to the manufacturer's settings will be documented during installation of GS2.

GS2 is not CE marked as a Medical Device and an application for a pre-market approval for the study described in this protocol has been submitted to the Competent Authority, Swiss Medic.

Caregivers (physicians/nurses) will be advised by GS2 on glycaemic control and nutrition management of patients in the intervention group. The caregivers will be instructed in the use of GS2.

A patient randomised to the intervention group must be created as a patient in GS2. To do this one of the investigators must logon to GS2, which ensures the authentication and authorisation. The investigator then types in the Centricity ID of the patient, which initiates a search for the ID in Centricity. When a match for the ID is found, the patient's demographic data are automatically imported from Centricity, including name, ID, age, gender, weight, height, diabetes, admission date and time.

Once the patient has been created, GS2 can support the workflow for two different tasks: 1) Setting the nutritional targets and 2) Advising on nutrition and insulin therapy. A summary of these two tasks is given below. Further detail can be found in Instructions for Use (GS2-IFU).

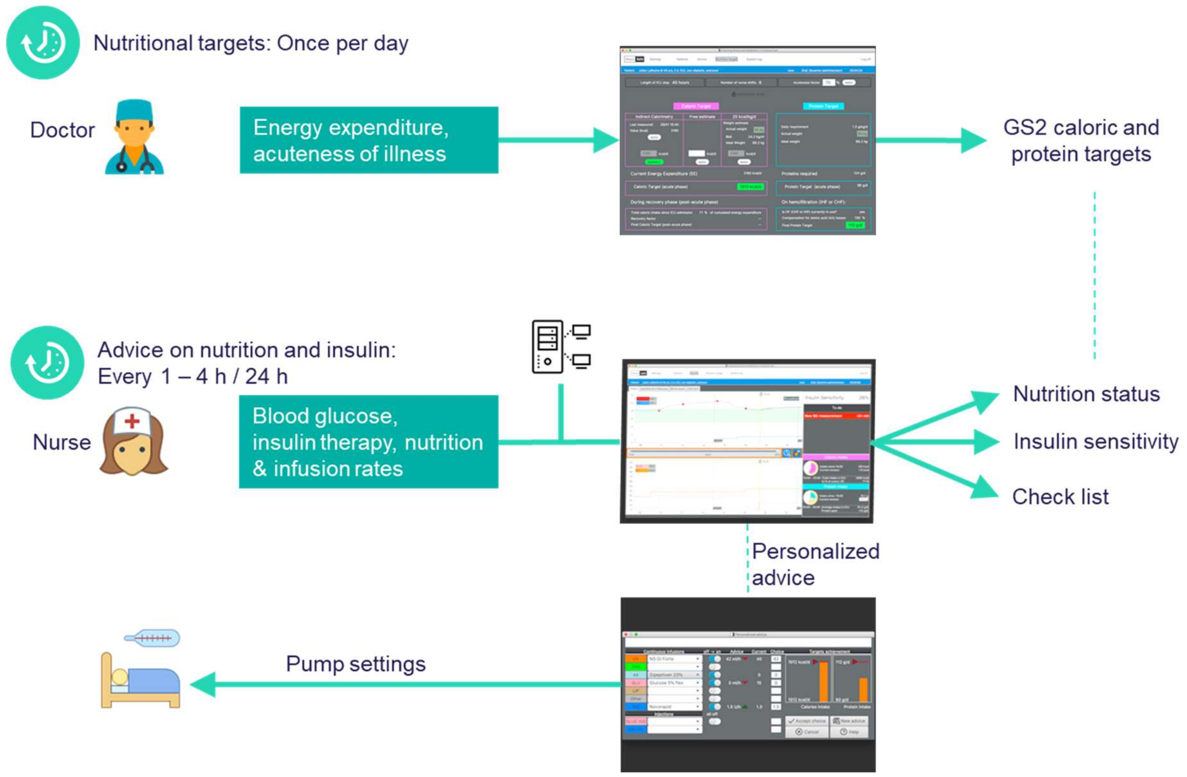

Figure5. The two workflows supported by GS2: Setting the nutritional targets and advising on settings of nutrition and insulin therapy pumps.

Setting the nutritional targets

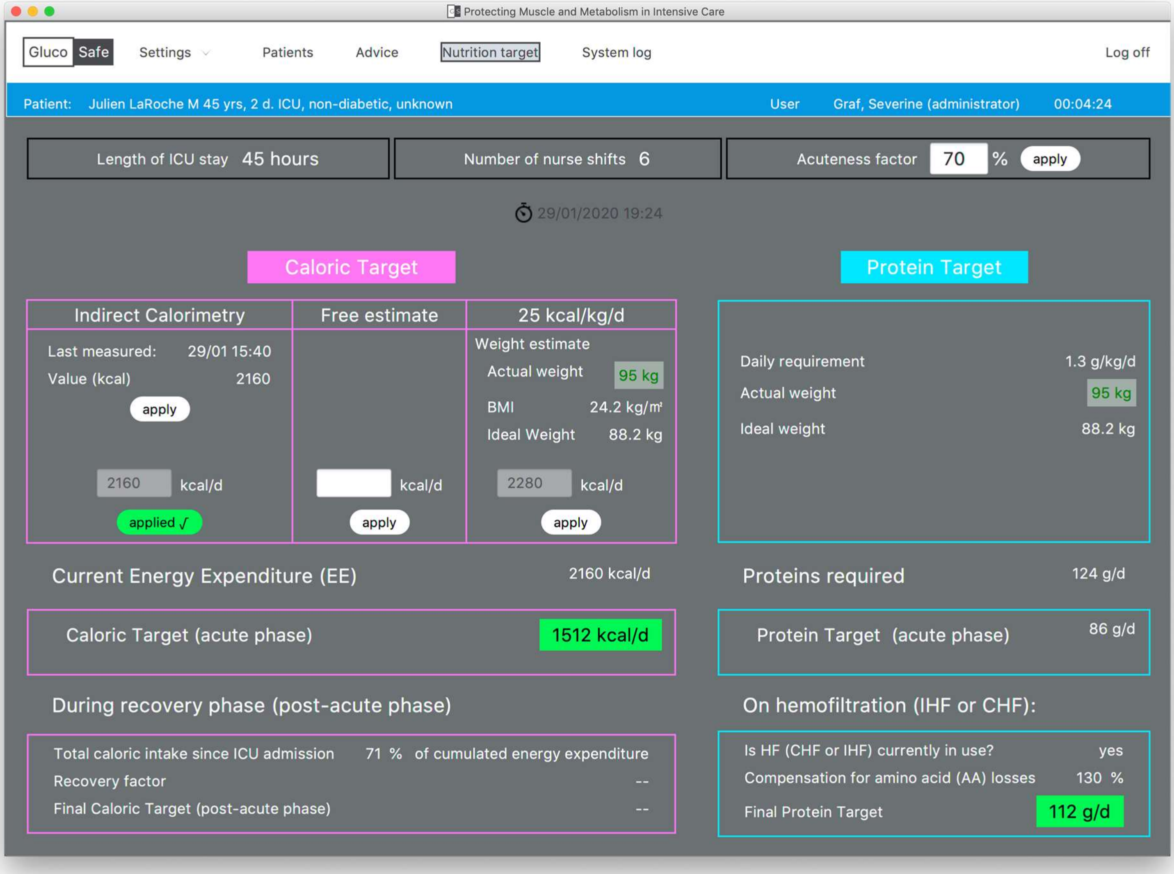

Figure 6. The page used in GS2 to set the nutritional (caloric and protein) targets.

Nutritional targets are set once every day by the attending physician. To set the nutritional targets the user must provide an estimate of the patient's Current Energy Expenditure (EE) and of the patient's Acuteness factor. The estimated EE is provided by clicking on one of the three "apply" buttons for Indirect Calorimetry, Free estimate or 25 kcal/kg/day. In the example in Figure 6. Indirect Calorimetry has been selected, resulting in an estimate of 2160 kcal/d for EE. The patient's Acuteness factor is provided by typing it (70%) in the Acuteness factor field and clicking "apply". The Acuteness factor is by default set to 20% at admission to the ICU and is automatically increased by 10% at the beginning of each nurse shift, until it reaches 100%. It can be set to any value between 0% and 100% at any time. The Caloric target is then calculated as the product of EE and the Acuteness factor (1512 kcal/d). The Proteins required (124 g/d) are calculated as the Daily requirement (1,3 g/kg/d) multiplied by the patient's weight (or ideal weight, if the BMI is above 25kg/m^2). The Protein target (86 g) is then calculated as the Protein Target multiplied by the Acuteness factor. The Final Protein Target (112 g/d) is the same as the Protein Target, unless the patient is on hemofiltration, in which case it is 130% of the Protein Target.

Advising on nutrition and insulin therapy.

Advice on insulin and nutrition is required every time a new BG measurement becomes available. On the GS2 "Advice" page the user is reminded when the next BG measurement is due. In the example in Figure 7, this is in -30 min, or in other words, the measurement is 30 min overdue. The user should respond by making a BG measurement and reporting it to Centricity. Within 10s GS2 will have imported the measured BG and will replace the "To do" item " BG measurement due" with a new "To do" item "Request new advice".

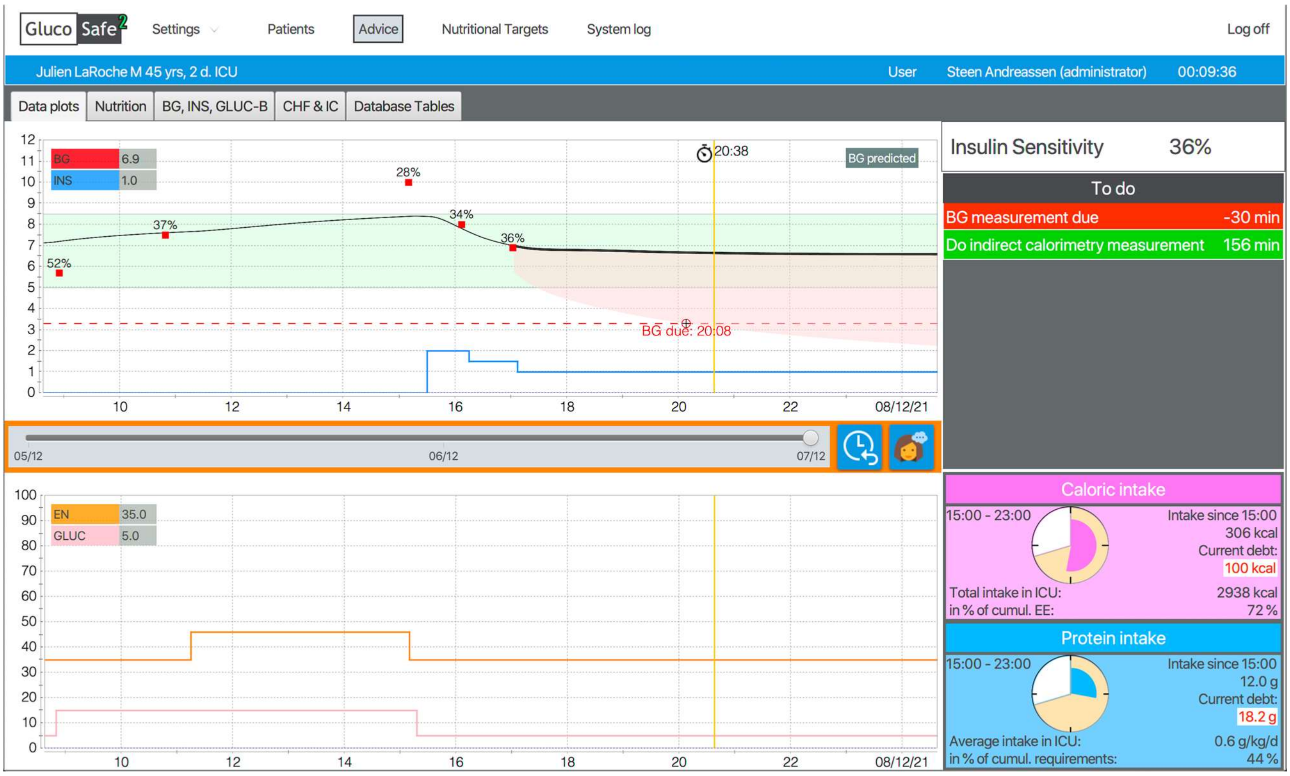

Figure7. The GS2 "Advice" page which is used to request advice. The page displays the history of insulin therapy, the BG measurements, and simulated and predicted BG (black curve) in the diagram at the top left. The nutrition history is displayed in the bottom left diagram. On the right-hand side, the upper panel shows the current insulin sensitivity of the patient, current "To do" items (user tasks) and when they are due or overdue. Finally, the lower right-hand panel displays the current state of achievement of the caloric and protein intake targets with respect to the ongoing nurse shift, and for the entire stay in the ICU.

The user can request advice by clicking the face icon on the middle-left bar. GS2 then shows the advice pop-up (Figure 8). In the example given below, GS2 advises to reduce enteral nutrition (Novasource® GI Forte) from the current rate of 45 ml/h to 35 ml/h. It also recommends starting an amino acid supplement in the form of 15 ml/h Dipeptiven® and stopping the glucose infusion. The advised insulin dose remains unchanged at 1 U/h. The right-side panels show that both the caloric and protein targets are being met. The user can choose to explore other treatment options, or click on "Accept Choice". The user must then set the pumps according to the advice and report the pump settings to Centricity.

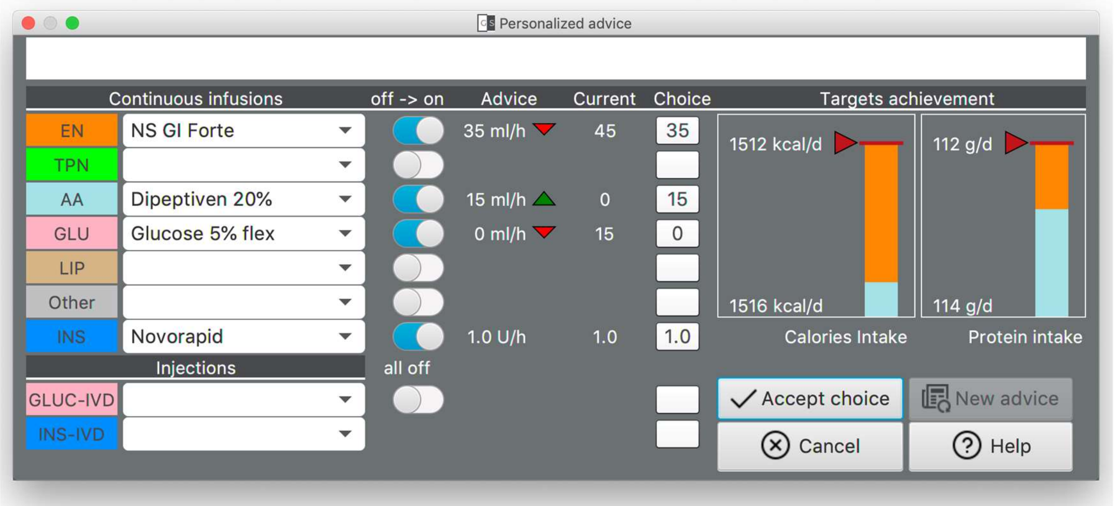

Figure 8. The GS2 Advice pop-up window, which displays the recommended pump rates for the nutrition and insulin pumps. The two panels on the right display the achievement of calorie and protein targets that were achieved in the figure.

Calculation of due time for the next blood glucose measurement

GS2 uses the BG prediction error to calculate the maximum time until the next BG measurement is due. The purpose of this is to keep the hypoglycaemia risk at any time below the acceptable risk maximum of 0.05% per measurement, or 2% per patient (under the assumption that one patient has on average about 40 measurements taken, 2% is given by 0.05% *40). The prediction error is a function of the prediction time, the longer the prediction, the larger is the "uncertainty" of the predicted BG, and thus the "uncertainty curve" has the shape of a "funnel", as can be seen in Fig. 7 above (light-pink shaded area beneath the thicker black line depicting the predicted BG in the upper left-hand plot).

A close-up of Fig. 7 with annotations in text is shown below. The time of the next measurement is when 1) the first time the lower edge of the uncertainty curve reaches the hypoglycaemia threshold, or 2) the next measurement is due according to local department rules, when that is sooner than 1).

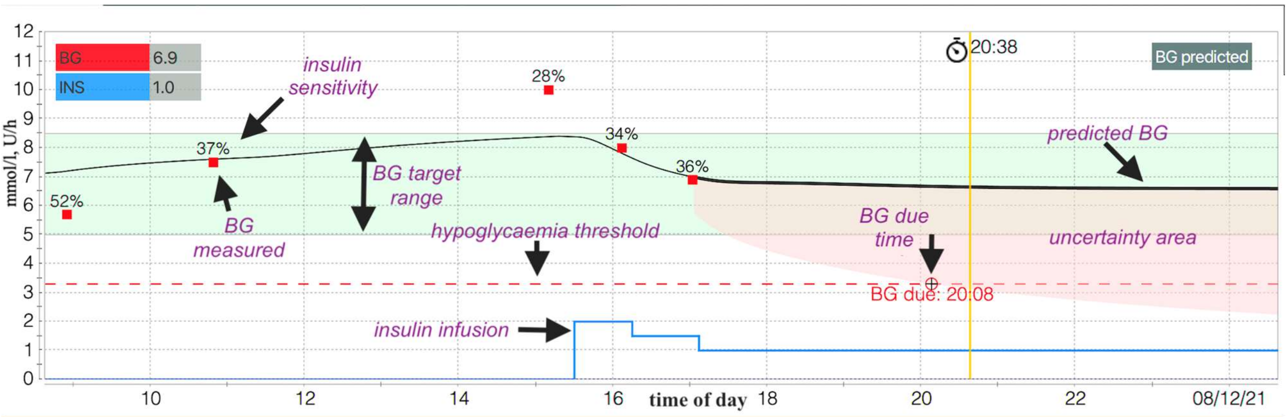

A close-up of Fig.7 upper left-hand panel: The next BG measurement is due at 20:08, when the lower edge of the uncertainty area (light-pink shaded) the first time touches the hypoglycaemia threshold (dashed horizontal line) after the most recent BG measurement (red squares). The BG target range is depicted as light-green area, and each BG measurement shows the insulin sensitivity that was calculated at the time of the measurement.

In mathematical terms, the "uncertainty curve" at each time t for the predicted BG concentration is:

$$UC(t) = \exp(\ln \text{predicted BG}(t) + \text{Norm.Inv}(0.0005) * \ln PE(t)$$

where 0.0005 or 0.05% is the "Hypoglycaemia Risk Maximum" setting, i.e., the highest risk of hypoglycaemia deemed acceptable. Norm.Inv(0.0005) = -3.29 is the inverse of the cumulative normal distribution.

The prediction error PE used to draw the uncertainty curve will be estimated from retrospective data analysis of the historical control group, and the prediction error curve will be drawn and compared to the graph shown in Fig.4. As explained in Section 3.4, the cut-off point to discontinue the trial has been set to the comparison of the graphs on the prediction time at 2 hours, which is expected to be the most frequent interval between BG measurements in hyperglycaemic patients in the acute phase. The rationale for stopping the study is that the uncertainty curve reaches the hypoglycaemia threshold earlier with a larger prediction error, and although the hypoglycaemia risk remains constant, the workload would increase.

8.1.2 Control Intervention (standard/routine/comparator)

The current local protocols for glycaemia control and nutrition management will be used in the control group and in the historical control group for comparison.

After ICU admission, a BG measurement is performed. If glycaemia is in the accepted range (5.0-8.5 mmol/l) or spontaneously normal (4.0 to 5.5 mmol/l), no correction is proposed. In this case, a BG control should be done every 2 hours. In case BG measurements are twice > 8.5 mmol/l, a new control is proposed in another sampling site. If the BG measurement is still > 8.5 mmol/l, an IV insulin-therapy should be initiated following the local protocol. A further BG control should be done within the next 15-30 minutes until stability of the BG. Then, BG controls should be done every 2 hours. If BG measurement are < 4 mmol/l, insulin-therapy should be stopped (if in progress) and a correction can be performed. A new control is done within 15 minutes. If the glycaemia is still under 4, a new correction is done. If the glycaemia is spent above 4, BG control are spaced to every 30 minutes. Then, when the glycaemia spent above 5, BG control are done every 2 hours. The algorithm of the local glycaemia control ICU protocol is presented in Figure 9.

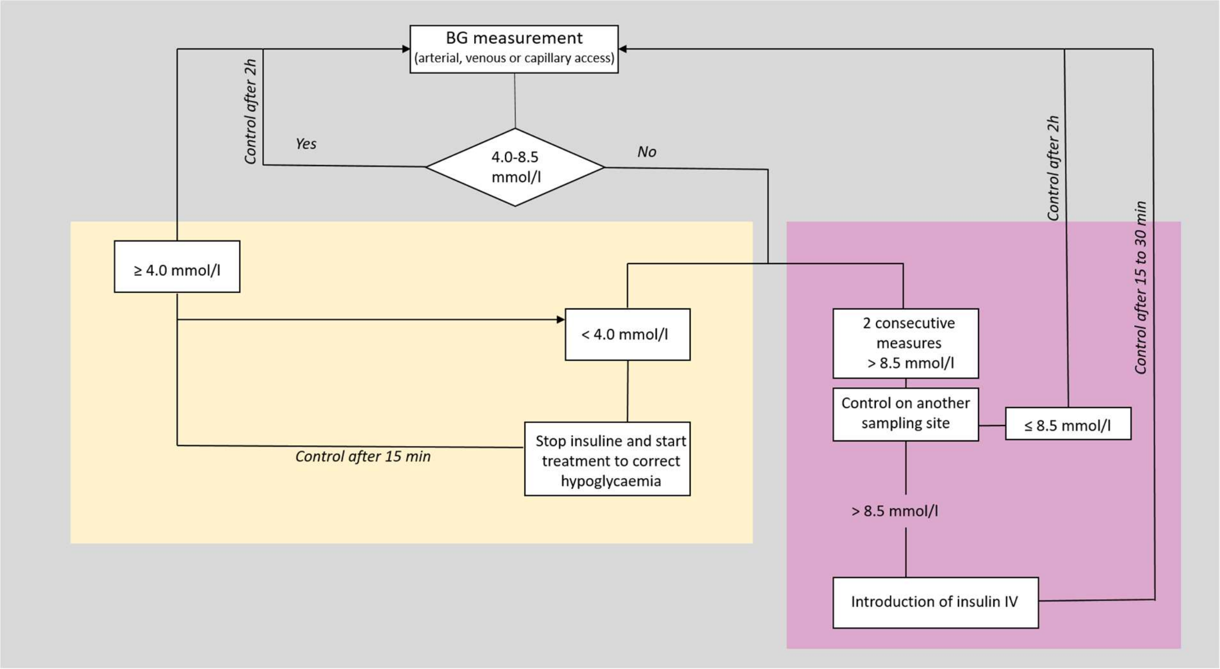

Figure 9. Algorithm of the local glycaemia control ICU protocol (control and historical control group)

An estimation of caloric target is performed after the admission in the ICU. Then, if the patient stays more than 4 days and if the indirect calorimetry measurement is achievable, the measurement is performed, and the caloric target is adjusted. If gastro-intestinal tract is functional and there are no contraindications to enteral nutrition, it is started from day 1 with an appropriate solution regarding the pathologies and with an initial debit of 25 ml/h or 10 ml/h for a patient with severe shock (can be adapted according to tolerance). The 80-100% of the energy target need to be achieved at the end of day 3. If the energy target (80-100%) is not covered at day 4, a SPN (Supplemental Parenteral Nutrition) is indicated to cover the target.

If enteral nutrition is contraindicated, a parenteral nutrition can be initiated in a progressive way. It can be either by a central venous access or by a peripheral one. Vitamins and trace elements should be supplemented with 1 ampoule of Addaven® and 1 ampoule of Cernevit®. Glutamine supplements can also be added on a case-by-case basis. If tolerance is good, PN is progressively increased to achieve 80-100% of the energy target by the end of day 3. The algorithm of the local ICU nutrition management protocol is presented in Figure 10.

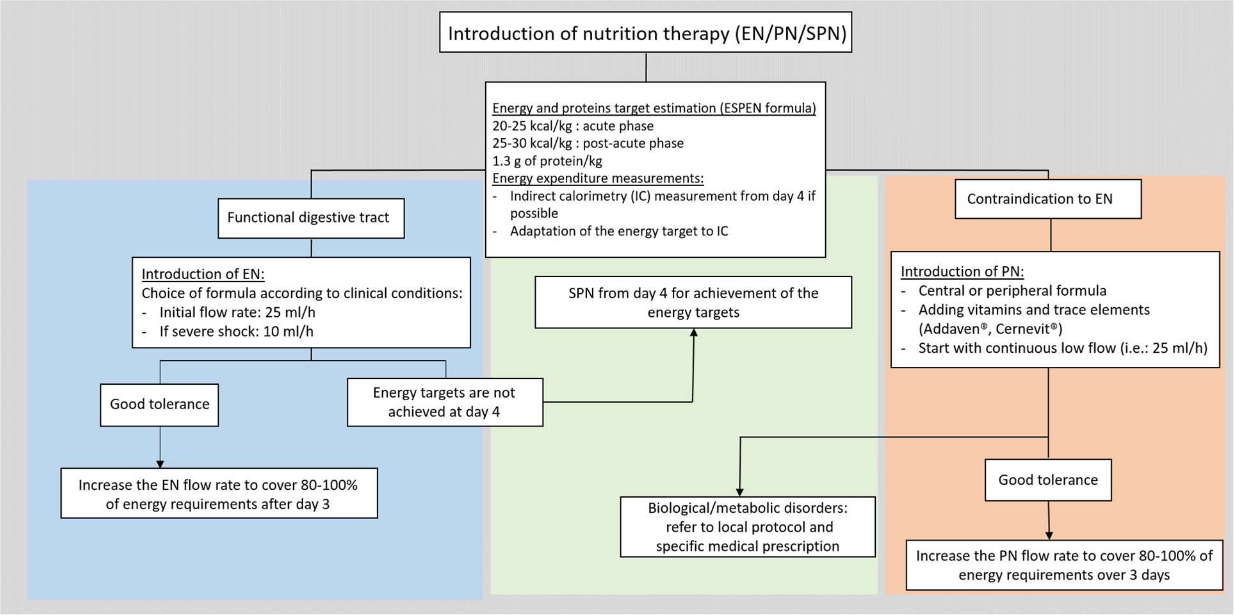

Figure 10. Algorithm of the local ICU nutrition management protocol (control and historical control group)

8.1.3 Labelling and Supply (re-supply)

GS2 will be installed in all bedside computers of the ICU by the IT manager of the ICU. The downloaded SW package will have the version number in the filename. Further information and labelling can be found on the GS2 Login page. A complete User Manual or Instructions for Use (IFU) will be supplied as a pdf-file.

8.1.4 Storage Conditions

Not applicable.

8.2 Discontinuation or modifications of the intervention

According to section 8.1.1 (Experimental Intervention (medical device)), the users of GS2 always have the option to accept GS2 advice, to accept it with modification of to refuse it. In this context, there are no situations in which the intervention will be discontinued or modified. As explained in point 7.4, the intervention of a patient is discontinued in case of death; withdrawal of life support; or consent withdrawal.

8.3 Compliance with clinical investigation intervention

Some strategies to improve adherence to the use of GS2 will be made.

Training: All caregivers will participate in training sessions before the beginning of the study. They will also receive a summary brochure with the main information. During the trial a clinical investigator will come daily to see if the caregiver has any question and if there is a need for a one-to-one training.

To do list: GS2 contains a “To do list” as a reminder for the caregivers to use the system. The non-compliance will not be analysed while the study is underway. The rate will be analysed at the end of the study as a secondary outcome and will be defined as:

- Number of episodes per patient and in the cohort where a BG measurement is not followed up within 30 minutes by a request for GS2 advice.
- Number of episodes per patient and in the cohort where pumps were not set within 30 min according to a GS2 advice accepted by the nurse (nurse did not notice, was busy, forgot, or misinterpreted advice = true errors)

8.4 Data Collection and Follow-up for withdrawn subjects

The medical follow-up of withdrawn subjects, or of subjects that drop out from the investigation prematurely is described in chapter 9.2.5 and chapter 9.2.6.

8.5 Clinical investigation specific preventive measures

No medications or treatments are prohibited during the study. The use of medications that play a role in glycaemic control will be recorded in the CRF.

8.6 Concomitant Interventions (treatments)

Apart from insulin therapy and nutritional therapy, all the medications and treatments will follow the usual practice in the ICU-HUG.

8.7 Medical Device Accountability

Installation of the GS2 software on HUG's bedside computer stations will follow the procedure described in the Investigator's Brochure. A record of every installation (one installation per bedside computer) is made (See Integration Test Report Template, Appendix 6.) and kept at HUG-ICU. A copy of the report and a copy of the installation log file from every PC will be sent to the Manufacturer prior to the start of the clinical investigation.

Uninstallation of the GS2 software on HUG's bedside computer stations will be conducted by the HUG-ICU IT-manager. A record of every uninstallation is made by updating the information in the Integration Test Report. A copy of the report will be sent to the Manufacturer after conclusion of the clinical investigation.

8.8 Return, Analysis or Destruction of the Medical Device

All data recorded in the central GS2 Database will be transferred in coded form to HUG's secuTrial® data repository. This transfer is done as the data becomes available or at the end of the study. Once the Monitor has completed its inspection, the IT manager of the ICU will delete the central GS2 Database and remove GS2 from the bed-side computers of the ICU-HUG.

In case of device deficiencies, including malfunction, usability issues, or inadequacy in the information supplied by the manufacturer including labelling, the log file will be sent to the manufacturer for analysis.

9. CLINICAL INVESTIGATION ASSESSMENTS

9.1 Clinical investigation flow chart(s) / table of clinical investigation procedures and assessments

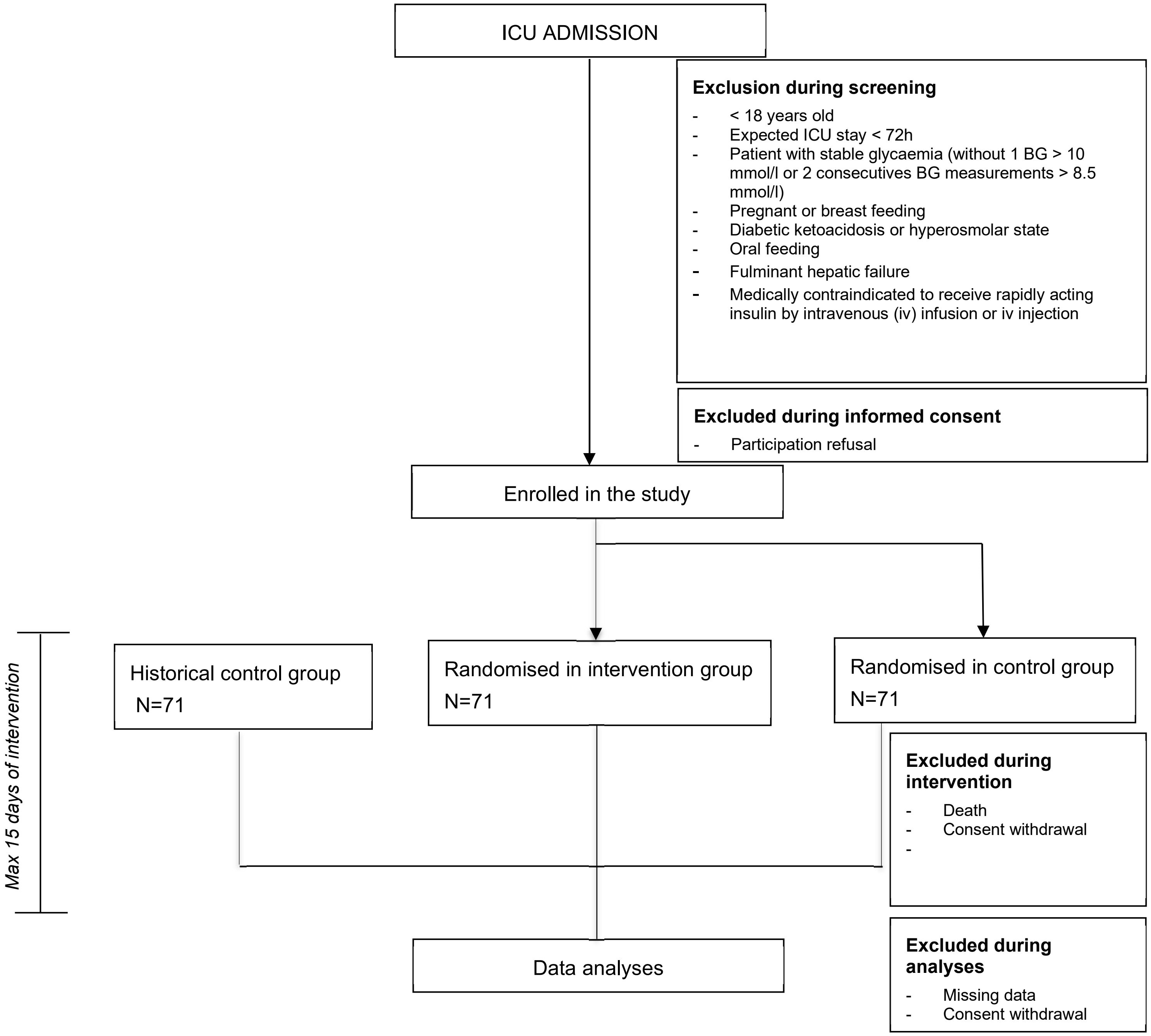

9.2 Assessments of outcomes

9.2.1 Assessment of primary outcome

Time-in-target (range: 5.0 to 8.5 mmol/l) per patient and in the cohort:

- All the BG measurement will be recorded with the time of the measurement in the eCRF. Then the time spent in the target (5.0 to 8.5 mmol/l) will be calculated in percentage for each patient and for each of the patient groups.

9.2.2 Assessment of secondary outcomes

Glycaemic “variability”:

Mean and SD of BG

- All BG measurement will be reported in the eCRF daily. The mean BG with SD will be calculated from those data.
- Maximum of daily BG difference

Achievement of nutritional targets.

The protein goal is met (e.g., 80-100% of the accumulated target)

- In the control group, the total intake of protein per day is going to be reported in the eCRF. Then, it is going to be compared with the daily target and the percentage will be made. The mean percentage for the duration of the study will then be calculated.
- In the intervention group, the comparison between the daily intake and the daily target is carried out automatically by GS2. The percentage will be reported directly in the eCRF. The mean percentage for the duration of the study will when be calculated.

The caloric goal is met (e.g., 80-100% of the accumulated target)

- In the control group, the total intake of energy per day is going to be report in the eCRF. Then, it is going to be compared with the daily target and the percentage will be made. The mean percentage for the duration of the study will when be calculated.
- In the intervention group, the comparison between the daily intake and the daily target will be made automatically by GS2. The percentage will be directly report in the eCRF. The mean percentage for the duration of the study will then be calculated.

Energy debt at the end of the stay

- In the control group, the total intake of energy per day is going to be reported in the eCRF. The daily energy target will be reported every day as well. Then the total energy debt is calculated as the addition of the daily difference between the energy target and the energy intake.
- In the intervention group, the energy debt will be directly calculated and extracted from GS2.

Protein debt at the end of the stay.

- In the control group, the total intake of protein per day is going to be reported in the eCRF. The daily protein target will be reported every day as well. Then, the total protein debt will be calculated as the addition of the daily difference between the protein target and the protein intake.
- In the intervention group, the protein debt will be directly calculated and extracted from GS2.

9.2.3 Assessment of other outcomes of interest

Workload:

Frequency of BG measurements per patient and in the cohort

- All the BG measurements will be daily reported in the eCRF. The mean number of BG measurements with standard deviation (SD) will be calculated per patient and in the cohort.

Frequency of adjustments of insulin and nutrition pump settings

- All the insulin adjustments will be daily reported in the eCRF. The mean number of insulin adjustments with SD will be calculated per patient and in the cohort.

Non-compliance (only in intervention arm)
Number of episodes per patient and in the cohort where a BG measurement is not followed up within 30 minutes by a request for GS2 advice.

- Data will be extracted for analysis from the GS2 database.

Number of episodes per patient and in the cohort where pumps were not set within 30 min according to a GS2 advice accepted by the nurse (nurse did not notice, was busy, forgot, or misinterpreted advice = true errors). Data will be extracted for analysis from the GS2 database.

Number of advices given by GS2 which were accepted, accepted with modification and rejected. Data will be extracted for analysis from the GS2 database.

Prediction error of BG
For each measured BG a prediction is made by GS2's metabolic model and for each of the following measured BGs the difference between natural logarithm of the measured and the predicted BG is calculated. The differences are placed in one-hour wide bins according to the prediction time. The prediction time is the time elapsed between the BG measurement from which the prediction is made and the later measured BGs. For each one-hour bin the root mean square of the logarithmic differences in that bin is plotted against the average prediction time in the bin. An example is given in the figure below from which it for example can be seen that the one-hour prediction error is about 14%. The curve which is fitted to the plot will be used as the BG prediction error and it is used to compare the quality of GS2 BG prediction with predictions by other metabolic models in the literature [35]. It is also used by GS2 to determine when the predictive error has become so large that a new BG measurement is required. The data required for the calculation of the BG prediction error will be extracted for analysis from the GS2 database.

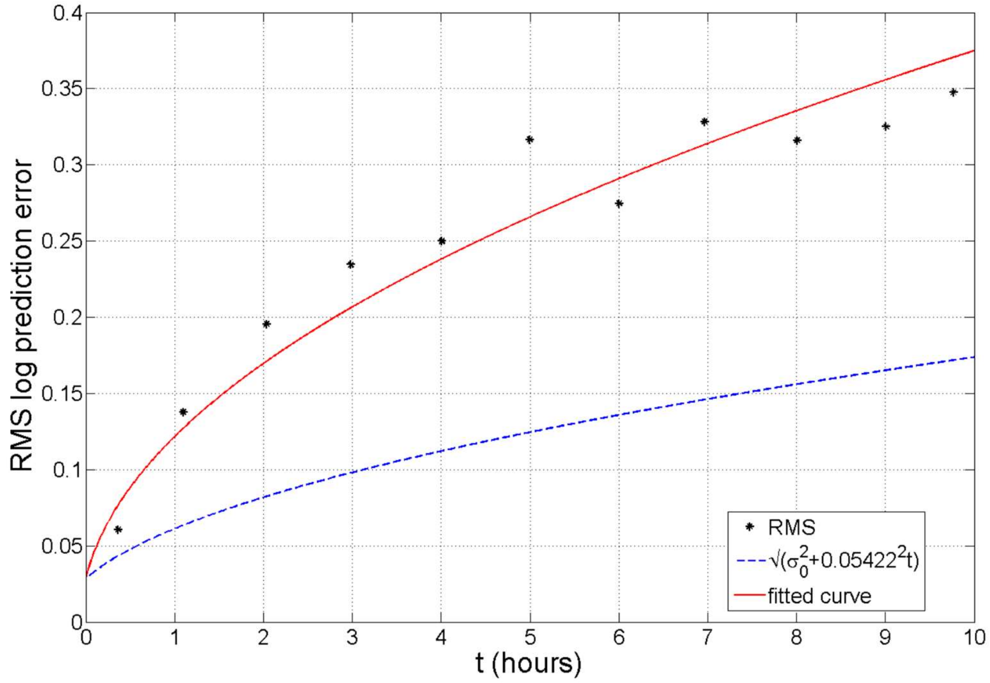

The Root Mean Square of the prediction error for each one hour prediction interval.

9.2.4 Assessment of safety outcomes

Hypoglycemia

- Overall number and percentage of mild (≤ 3.2 mmol/l) and severe (≤2.2 mmol/l) hypoglycaemic events per patient and in the cohort
  - All hypoglycaemia episodes will be reported in the eCRF, number and percentage will then be calculated.

- Number and percentage of mild (≤3.2 mmol/l) and severe (≤2.2 mmol/l) hypoglycaemia events due to non-compliance (only in intervention arm)
  - All hypoglycaemia episodes will be reported in the eCRF. Then, those data will be analysed jointly with the "non-compliance" outcomes.

Hyperglycemia (> 8.5 mmol/l)
- Time to normalize blood glucose (the first two consecutive values values ≤ 8.5 mmol/l as indicator for normalization)
  - All the BG measurements will be reported in the eCRF with the time of the measurement. The time to normalize BG will be analysed from those data for the control and the intervention group.
- Percentage of time in the ICU with hyperglycaemia (BG > 8.5 mmol/l) before and after normalization per patient and in the cohort
  - BG values are considered constant from one measurement to the next. Percentage of time in the ICU with hyperglycemia is defined as cumulative time with BG > 8.5 divided by the time spent in the ICU.
- Percentage of time in the ICU with hyperglycaemia (BG > 8.5 mmol/l) before and after normalization per patient and in the cohort due to non-compliance (only in intervention arm)
  - Percentage of time with hyperglycemia will be analysed jointly with the "non-compliance" outcome.
- Number of hyperglycaemic episodes after normalization per patient and in the cohort
  - All hyperglycaemic events will be record in the CRF. Those data will be analysed jointly with the "Time to normalize BG" outcome.
- Number of hyperglycaemic episodes after normalization per patient and in the cohort due to non-compliance (only in intervention arm)
  - All hyperglycaemic events will be record in the CRF. Those data will be analysed jointly with the "Time to normalize BG" and the "non-compliance" outcome.

Adverse events

Collected AE are detailed in point 10.3.1.

Vital signs

Monitoring of the vital signs will stay the same as the routine monitoring during the study. The blood pressure, temperature, heart rate, respiratory rate, ventilation mode and the presence of CVVHDF and ECMO will be report daily in the eCRF.

9.2.5 Assessments in subjects who prematurely stop the clinical investigation

For the patients prematurely withdrawn of the study, no follow up is planned. The data collected so far will be destroyed and will not be analysed (OClin Art. 17, alinea 3) [8] (see point 2.7 for more details).

9.2.6 Follow-up of the subjects after the regular termination of the clinical investigation

Only duration of ICU stay, MRC score at ICU discharge, alive status and BMI at hospital discharge will be reported. No other follow-up is planned.

9.3 Procedures at each visit

9.3.1 Screening visit

Screening will be done once a day (Monday to Friday) with the help of the screening form. Once the screening is done, study information will be given to the includable patients or to the legal representative and a physician who is independent to the study. If the patient consent to participate to the study, he will be randomized in either the control or the intervention group. Because of the large number of patients, only the included patients will receive a study number.

9.3.2 Inclusion visit

This visit will take place after written consent is obtained and randomization is done. The baseline data (from ICU admission) will be retrospectively reported in the eCRF form. Data included are: demographics and anthropometrics, date and time of hospitalisation and admission in the ICU, primary diagnosis and known chronic illness, medication as indicated in synopsis, laboratory, vitals and severity

score. The control or intervention will then start as explained in session 8.1.1 and 8.1.2.

9.3.3 Daily visit

Daily manual data as well as AEs will be reported in the eCRF will be reported during daily visits.

9.3.4 Upon ICU leave visit

Duration of ICU stay, MRC score at ICU discharge, alive status and BMI at hospital discharge will be reported in the eCRF.

10. SAFETY

Device deficiencies, AEs and SAEs collected, are fully investigated and documented in the safety report form and appropriate case report form (CRF) during the entire study period (from patient's informed consent until the end of the ICU stay or the end of the 15 days of follow up) [ISO 14155]. Documentation includes date of the event, treatment, resolution, assessment of seriousness and causal relationship to device and/or study procedure.

10.1 Definition and Assessment of (Serious) Adverse Events and other safety related events

Adverse Event (AE) (Art. 2 Abs 57 MDR) [5]

Any untoward medical occurrence, unintended disease or injury or any untoward clinical signs (including an abnormal laboratory finding) in subjects, users or other persons whether or not related to the MD.

Serious Adverse Event (SAE) (Art. 2 Abs 58 MDR) [5]

Any adverse event that led to any of the following:

- (a) death,
- (b) serious deterioration in the health of the subject that resulted in any of the following:
  - (i) life-threatening illness or injury,
  - (ii) permanent impairment of a body structure or a body function,
  - (iii) hospitalisation or prolongation of patient hospitalisation,
  - (iv) medical or surgical intervention to prevent life-threatening illness or injury or permanent impairment to a body structure or a body function,
  - (v) chronic disease,
- (c) foetal distress, foetal death or a congenital physical or mental impairment or birth defect.

Note: planned hospitalization for pre-existing condition, or a procedure required by the CIP, without a serious deterioration of the health status of the subject, is not considered an SAE.

Device deficiency (Art. 2 Abs 59 MDR) [5]

Inadequacy of a medical device related to its identity, quality, durability, reliability, safety or performance, of an investigational device, including malfunction, user errors and inadequate information supplied by the manufacturer.

Events such as malfunctions of the software in the automatized extraction data, in the glycaemia prediction, in the insulin sensitivity calculation or in the advice given will be reported as device deficiency.

Malfunction (ISO14155) [2]

Failure of an investigational device to perform in accordance with its intended purpose when used in accordance with the instructions for use or the CIP.

Device deficiency with Serious Adverse Device Effect (SADE) potential (Art. 80 Abs 1 letter c MDR; ISO14155) [2]

Any device deficiency that might have led to a serious adverse event if appropriate action had not been taken, intervention had not occurred, or circumstances had been less fortunate.

Adverse Device Effect (ADE) (ISO14155) [2]

Adverse event possibly, probably or causally related to the use of an investigational device or procedures.

Serious Adverse Device Effect (SADE) (ISO14155) [2]

Adverse device effect (ADE) that has resulted in any of the consequences characteristic of a serious adverse event.

Unanticipated Serious Adverse Device Effect (USADE) (ISO14155) [2]

Serious adverse device effect (SADE) which by its nature, incidence, severity or outcome has not been identified in the current version of the risk analysis report.

Causal Relationship of SAE (MDCG 2020-10/1) [36]

A causal relationship towards the medical device or the procedure of the investigation should be rated by the PI and the Sponsor as follows:

- **Not related:** The relationship to the device or procedures can be excluded.
- **Possible:** The relationship with the use of the investigational device is weak but cannot be ruled out completely. Alternative causes are also possible.
- **Probable:** The relationship with the use of the investigational device seems relevant and/or the event cannot reasonably be explained by another cause.
- **Causal relationship:** The serious event is associated with the investigational device or with procedures beyond reasonable doubt.

10.2 Adverse events categorization

The adverse events are categorized by the PI and the Sponsor using the following algorithm:

Does the AE meet the seriousness criteria?

- No, it is not serious
    - Is the relationship to the device or the procedure possible, probable or causal?
        - No: non-related AE
        - Yes: ADE
- Yes, it is serious: SAE
    - Is the relationship to the device or the procedure possible, probable or causal?
        - No: non-related SAE
        - Yes: SADE
- Is it anticipated (within expected type, severity and frequency of the complications)?
        - No: unanticipated SADE (USADE)
        - Yes: anticipated SADE (ASADE)

10.3 Documentation and reporting in Medical Device Category C clinical investigations

Device deficiencies and all **adverse events (AE)** including all **serious adverse events (SAE)** are collected, fully investigated and documented in the safety report form and appropriate CRF during the entire investigation period, i.e. from patient's informed consent until the last CIP-specific procedure, including a safety follow-up period.

- Documentation of AEs (including SAEs) by the PI includes diagnosis or symptoms, start and stop

dates of event, event treatment, event resolution, assessment of seriousness and causal relationship to MD and/or investigation procedure (Art. 32 ClinO-MD, ISO14155) [2, 4].

- Documentation of DDs by the PI includes description of event, start date, investigational device information, action taken regarding the investigational device, and whether the DD led to an AE. The Sponsor shall review all DDs and determine and document in writing whether they could have led to a SAE (DD with SADE potential) (Art 32. ClinO-MD, ISO14155) [2, 4].

The Sponsor provides the CA and the CEC with the documentation at their request (Art. 32 ClinO-MD) [4]. AEs which can be linked with the study (see point 10.3.1) will be assessed during the daily visit.

10.3.1 Foreseeable adverse events/anticipated adverse device effects

As this is the first study to evaluate the safety and performance of the GS2 device in humans, we do not yet know the likely incidence of the anticipated adverse device effects. Therefore, we have listed the anticipated ADEs below, based on the risk management report performed in accordance with ISO 14971:2019. The probability of occurrence of the hazardous situation and the probability of severity level have been estimated in accordance with the residual risk assessment of the risk management report.

**Anticipated ADEs**	**Probability of Severity Level (S)**	**Probability of Occurrence of a Hazardous situation (O)**
Metabolic and nutritional disorders		
Hypoglycemia event (≤3.2 mmol/l)	Negligable to critical (S1-4)	Remote to Frequent (O2-5)
Hyperglycemia event (≥ 8.5 mmol/l)	Negligable (S1)	Remote to Occasional (O2-3)
Malnutrition: Short-term over-nutrition	Minor to Serious (S2-3)	Occasional (O3)
Malnutrition: Short-term under-nutrition	Minor to Serious (S2-3)	Probable (O4)
Malnutrition: Over-nutrition	Minor (S2)	Occasional (O3)
Malnutrition: Under-nutrition	Minor (S2)	Occasional (O3)
Gastrointestinal disorders		
- Digestive disorder such as vomiting, diarrhea	Minor to serious (S2-3)	Occasional (O3)

For all ADEs listed above, risk mitigation was performed as described in the risk management report. For example, for hypoglycemia, the interval until the next BG measurement was shortened in certain situations. In addition, the maximum increase in insulin dose was limited, and some written comments were made to raise user awareness.

10.3.2 Reporting of (Serious) Adverse Events, device deficiencies, and other safety related events

Procedure for the user to report unexpected/alarming results or problems:

This procedure has been described in the “Instruction for use” (GS2-IFU_V1.1). In case of unexpected/alarming results or problems, one of the study investigators will analyze the situation and fill out the device deficiency form if needed and inform the principal investigator (PI) of the study. The PI will decide whether the device deficiency requires a suspension of the study until the manufacturer has taken preventive or corrective actions. All information about the device deficiency will be collected and the manufacturer will be notified within 24 hours of working days. The manufacturer assumes the responsibility to carry out necessary steps and corrective measures.

Suspected device deficiencies fall into one of the following 4 categories:

1) Unexpected behavior/suspected errors
2) Advice that is not compatible with departmental guidelines
3) Slow performance
4) Malfunction/ reliability issues

In general, if a device deficiency is observed or suspected, one of the investigators should be contacted not later than 12 hours after the observance.

It is recommended, that the user acts according to the following schema:

Suspected device deficiency	Procedure
1) Unexpected behavior/suspected errors:<br>Examples:<br>- No new nutritional target is calculated by GS2, even though the Nutritional Targets page was just visited by the user<br>- Entering the ID of a patient into GS2 prompts GS2 message that "The ID cannot be found" even though the ID is known to be correct<br>- GS2 notifies the user that there is a new Indirect Calorimetry measurement available when no indirect calorimetry was done recently	Take a note of the situation (which patient, date & time of the observed behavior, description of the behavior, screenshot of GS2 if possible).<br>The user should discuss the note with one of the investigators during the daily visit.<br>The PI will decide if it is a device deficiency.
2) Advice that gives rise to concern:<br>Examples:<br>- Insulin dose (> 0 U/h) is advised when blood glucose is below target (5 - 8.5 mmol/l) and the patient is not insulin-dependent chronically diabetic.<br>- Glucose bolus injection is advised when patient's blood glucose is not hypoglycaemic.<br>- No nutrition advised for a patient on the fourth day in the ICU.<br>- GS2 advises to increase insulin in a single step larger than 2 U/h.	**Refuse the advice given by GS2 until the PI or co-investigators have been consulted**.<br>Take a note of the situation (which patient, date & time of the observed behavior, description of the behavior, screenshot of GS2 and of the advice window).<br>Contact the investigator immediately, not later than 12 hours after the observance.<br>The PI will decide if it is a device deficiency and if a suspension of the study is required.
3) Slow performance<br>Examples:<br>- Waiting time after a requested advice exceeds 20s until advice window opens.	Slow performance can be expected if a large amount of data of the patient has to be loaded, but it could also be either a device deficiency, or a failure of the external patient data management system Clinisoft.

- Waiting time to load a new patient from Clinisoft exceeds 2 minutes.<br><br>Waiting time for "Nutritional Targets" page, "Advice" Page, and "Patients" page exceeds 30s	Take a note of the situation (which patient, date & time of the observed behavior, description of the behavior, screenshot of GS2 if possible). Please add a description of how you interacted with GS2 and what the approximate time of waiting was.<br><br>The user should discuss the note with one of the investigators during the daily visit.<br><br>The PI will decide if it is a device deficiency.
4) Malfunction/technical issues:<br>Examples:<br>- Import of the patient's data from Clinisoft is incomplete (data shown in Clinisoft are not visible in GS2)<br>- Data "out-of-range" messages appear when entered data are correct, for example "75 kg is not a correct body mass"; "2345 kcal is not a correct energy expenditure"<br>- Graphical user interface freezes and inhibits use of GS2	The user should stop using GS2 until the investigator has been informed.<br><br>Meanwhile, take a note of the situation (which patient, date & time of the observed behavior, description of the behavior, screenshot of GS2 if possible).<br><br>Contact an investigator immediately.

Reporting to the Sponsor:

Reporting to Sponsor-Investigator:

The following events are to be reported to the Sponsor-Investigator <u>within 24 hours</u> upon becoming aware of the event:

- All reported SAEs
- Health hazards that require measures
- Device deficiencies

The Sponsor-Investigator will evaluate SAEs regarding causality and seriousness. Device deficiencies are assessed regarding their potential to lead to an SAE. The Sponsor will decide if the device deficiency needs a suspension of the study until a preventive or corrective action has been taken by the manufacturer (refer to GS2-IB, point 5.1).

Reporting to the manufacturer:

According to the contract regarding risk management, any device deficiency will be reported by the PI to the manufacturer. An electronic notification will be sent directly to the manufacturer when a device deficiency form is entered in the eCRF in secuTrial®. The PI will then confirm the good reception of the notification with the manufacturer within 24 hours. The manufacturer undertakes the responsibility to perform necessary updates to risk analysis, to the device itself, to the GS2-IB and to inform the Sponsor-Investigator of any corrective action taken.

Reporting to the Competent Ethics Committee and to Swissmedic:

Reporting of events to the Competent Ethics Committee and to Swissmedic will be subcontracted to the pharmacovigilance team of the UIC (Unité d'Investigation Clinique), a unit of the CRC (Centre de Recherche Clinique/CTU) at HUG.

The following events are to be reported to the CEC and to the CA promptly (Art. 33 ClinO-MD) [4]:

a. any serious adverse event which has a causal relation with the MD, comparator or procedure/test method or where a causal relation is probable or possible (SADE);
b. any device deficiency which, in the absence of appropriate measures or intervention or in less favourable circumstances, could have led to serious adverse events (DD with SADE potential);
c. any new information relating to an event already notified under points (a) and (b).

To ensure prompt notification, the Sponsor may initially submit an incomplete notification.

If safety and health hazards that require measures must be taken immediately during the conduct of the investigation, the Sponsor notifies the CEC within 2 days of these measures and the circumstances which made them necessary (Art. 34 ClinO-MD) [4].

Periodic safety reporting (Art. 35 ClinO-MD) [4]:

An Annual Safety Report (ASR) is submitted by the Sponsor to the CEC and to the CA, yearly (Art. 35, 38 ClinO-MD) [4]. The ASR contains a list of all SADEs and DDs and a report on their degree of seriousness, causal relationship with the MD and procedure and on subjects' safety.

10.3.3 Follow-up of (Serious) Adverse Events

All patients with reported AE or SAE will be followed during the entire study period (from patient's informed consent until the end of the ICU stay or the end of the 15 days of follow up) [ISO 14155]. SAEs will be followed until resolution or stabilization. Participants with ongoing SAEs at study termination (including the closing visit) will be further followed up until they recover or until stabilization of the disease.

11. STATISTICAL METHODS

11.1 Hypothesis

The null hypothesis is that the mean time in target glycaemia (defined as BG between 5 and 8.5 mm/L) will be equal between patients randomized in the intervention arm and in the standard of care arm (control group).

Alternative hypothesis is that the mean time in target glycaemia will be different between patients randomized into the intervention group and into the standard of care group (control group).

11.2 Determination of Sample Size

Based on data collected routinely at the ICU between January and March 2017, the time in target was 68% (SD 21%) for patients under the standard of care. Expecting the intervention to give an improvement of 10% of time in target, we would need 71 patients in each patient group to detect give the trial a power of 80% and an alpha error of 5% (two-sided). If the patient refuses to participate afterwards or withdraws from the study for whatever reason, they will be replaced in order to have 71 per group at the end of the study.

11.3 Statistical criteria of termination of the investigation

Patients will be followed until the end of ICU stay or before, if the standard of care naturally ends.

11.4 Planned Analyses

Continuous variables will be presented according to the study group by their mean (±standard deviation SD), median, interquartile range and range. Categorical variables will be presented according to the study group by their frequencies and relative proportions.

11.4.1 Datasets to be analysed, analysis populations

The dataset from intervention group will be analysed against both the control group and the historical control group. To assess a possible “cross-over” effect, the control group will be analysed against the historical control group. All analyses will be performed in an intention-to-treat basis: all patients will be analysed as randomized.

11.4.2 Primary Analysis

The primary outcome (time in target at the end of ICU stay) will be compared between the two groups using Student t test or Mann-Whitney nonparametric test, in case of non-normal distributions. Primary analysis will be done using linear regression with time in target as the dependent variable, the study arm as the main independent variable, and stratification variables (APACHE II categories and diabetic status) included as covariates (ref Points to consider on adjustment for baseline covariates. EMEA Agency, 2003). We will conclude superiority of the GS2 intervention, compared to the control or historical control groups, if the time in target is significantly higher ($p < 0.05$).

11.4.3 Secondary Analyses

For secondary outcomes, we will apply multiple regression models with adjustment on stratification variables and some prespecified variables.

- All continuous variables will be compared between the two study groups using either Student t test or Mann-Whitney nonparametric test; categorical variables will be compared using either Chi-2 test, or Fischer exact test, depending on application criteria.
- Count data (number of hyperglycemic episodes) will be compared using Poisson regression model or if data are over-dispersed, negative binomial regression models.
- Binary outcomes (protein goal achievement, caloric goal achievement) with repeated measurements will be analyzed using mixed-effects logistic regression models.
- Continuous outcomes with repeated measurements will be analysed using mixed-effects linear regression models.

11.4.4 Interim analyses

No interim analyses are planned.

11.4.5 Deviation(s) from the original statistical plan

If the statistical methods described herein prove unsuitable during the analyses, more appropriate methods will be used. All changes in methodology will be documented in the clinical study report.

11.5 Handling of missing data and drop-outs

Prior to analysis, any missing data will be investigated and reasons for missing data obtained and summarised where possible. All attempts will be made to avoid any missing outcome data and to correct for it. We do not plan to use specific methods for handling of missing data.

12. QUALITY ASSURANCE AND CONTROL

To ensure the quality of data, the PI will train the study staff on all important study-related aspects and will ensure that the study is performed according to the protocol, ICH-GCP and regulations.

12.1 Data handling and record keeping / archiving

12.1.1 Case Report Forms

All study data will be entered by a member of the research team directly into the secuTrial® platform except automatic data exportable directly from Glucosafe 2. SecuTrial® platform allows full auditing and traceability of modifications. Solely the research team and the PI will have access to the platform after a secure authentication.

Members of the research team authorized by the PI to enter the data in the eCRF will be listed on the delegation log.

All data will be pseudonymised in the secuTrial® platform. The subject identification list will be secured by a password and will be kept into a secured storage of the HUG.

12.1.1.1 <u>Electronic CRFs (eCRF)</u>

Last applicable version of the following documents:

- Printout e-CRF_prospective part secuTrial
- Printout e-CRF_retrospective part secuTrial
- Template automatic e-CRF_exported form

12.1.2 Specification of source data and source documents

The source documents are:

Data	Type of source
Eligibility Criteria	Hospital electronic medical record
Signed informed consent form	Original paper
Randomization	eCRF
Baseline data	Hospital electronic medical record
Daily manual data	Hospital electronic medical record
Daily automatic data	Electronic extraction from GS2
Upon ICU leave data	Hospital electronic medical record
Adverse event/Serious Adverse Event	eCRF
Subject withdrawal/ Lost of follow up	Hospital electronic medical record
Device deficiencies	Electronic extraction from GS2/eCRF

12.1.3 Record keeping / archiving

All study data must be archived for a minimum of 15 years after study termination or premature termination of the clinical trial.

12.1.4 Archiving of essential clinical investigation documents

All the documents of the investigation must be archived for a minimum of 15 years after regular or premature termination of the investigation. The source paper documents will be stored in a locked location on site. The eCRFs will be archived in secuTrial® platform of the HUG. An extraction of the pseudonymised database will be shared with AAU. In case the Glucosafe2 Medical Device obtains a CE marking, then the coded data will be kept for the duration of the lifetime of Glucosafe as a Medical Device, as required by CHAPTER VII, SECTION 1 "Post-market surveillance" Article 83 of the **REGULATION (EU) 2017/745** OF THE EUROPEAN PARLIAMENT AND OF THE COUNCIL of 5 April 2017 on medical devices, amending Directive 2001/83/EC, Regulation (EC) No 178/2002 and Regulation (EC) No 1223/2009 and repealing Council Directives 90/385/EEC and 93/42/EEC.

12.2 Data management

12.2.1 Data Management System

Data management will be performed by UIC (Unité d'Investigation Clinique), a unit which is part of the CRC (Centre de Recherche Clinique / CTU) at HUG and the Faculty of Medicine UniGE (Geneva University). UIC is certified ISO 9001/2008, and the unit guarantees best practices in the field of clinical data management. Data are physically stored in a [Oracle ver.12c/19c] RDBMS (Relational Database Management System) using a dedicated CDMS software (Clinical Database Management System) [secuTrial, a certified GCP-compliant electronic clinical data management system ®].

12.2.2 Data security, access and back-up

Physical access to the data centres is logged and limited to authorised personnel using badge authentication. On a regular basis, vulnerability testing is performed to reduce potential exposure. Remote access to servers is limited to authorised personnel. Connections to servers are encrypted using SSH. System logs are stored in a dedicated centralised system for audit purposes. The internal HUG network is protected by multiple firewalls, proxy, reverse-proxy and anti-virus solutions. Web servers operate under SSL (HTTPS) certifications, ensuring Web connections are encrypted and secure. At the CDMS level, only people part of the investigation team, the sponsor team, the affiliated reviewers or auditors, as well as inspection authorities (Swissmedic) are given access to data. Personal accounts are granted individually for each person. Identification is made by a personnel ID and a password. Failure to provide the correct password after a limited number of attempts automatically deactivates the faulty account (protection against non-authorized attacks). Only institutional e-mail addresses will be accepted for any communication of sensitive data regarding account creation and management. Pen-Tests (simulation of malware attacks) are regularly performed, and measures taken whenever necessary.

Data transfer: Exports of all or any kind of partial data will be systematically password encrypted before being transferred. Use of hashing encoding will ensure that no data alteration may have occurred during the transfer.

All data and applications are physically stored in dedicated data centres on HUG premises. The physical hardware consists of enterprise-grade servers, networking, and storage solutions from tier 1 vendors and trustworthy and stable GNU/GPL solutions.

Data are physically stored in a [Oracle ver.12c/19c]. RDBMS (Relational Database Management System) using a dedicated CDMS software (Clinical Database Management System) [secuTrial®]. This CDMS is a central Web-based system consolidating all CRF related data.

All applications are hosted on certified virtual servers running Red Hat Linux Enterprise in a VMWare ESX environment. There are several dedicated servers for each system, ensuring separation between the testing / pre-production environment and the production environment. System updates are applied only after validation in the pre-production environment.

For security reasons, the application tiers and the database management systems run independently in separate servers.

HUG infrastructure is under the responsibility of DSI (Direction des Systèmes d'Information at HUG). All exploitation, monitoring and backups operations are performed by DSI, in accordance with UIC policies.

All systems and applications are continuously monitored. Appropriate measures are automatically taken whenever an alert is issued. Backups operations are performed by the DSI service at HUG, in accordance with UIC policies. Frequent backups are performed using the best enterprise backup solutions at HUG and are physically stored in a fire-proof safe.

Backup strategy compromises an optimised hourly, daily, monthly and yearly retention plan:

- Server backups (DSI): 1x differential per day (24 days retention), 1x full every 24 days (retention 12 months), annual (preserved infinitely)
- Database backups (DSI): every day, with 2 full backups twice per week (double copies, 2 months retention for Sunday backups, and 14 days for the others), log backups every hour (2 months retention)

12.2.3 Analysis and archiving

At the end of the project, the entire database will be archived in a reusable format. Archives encompass all raw data, meta-data, transformed data, transformation operations, deviations, version history, and audit trails. Redeployment of the entire database is therefore possible whenever needed. The comprehensive archive will remain property of the sponsor and will be preserved during a minimum period of 15 years. UIC will also ensure an electronic copy of the archive will be stored within the DSI infrastructure for a theoretical infinite period of time.

Data will be provided to authorised third parties as much as possible in non-proprietary formats (text, CSV, XML, PDF).

The data management environment allows for specific role distinction, personal identification of all authorized users, personalization of permissions, rule-based data validation, automatic up-to-date reporting, data safety, monitoring and multi-level reviewing. Any action or intervention is recorded within a detailed Audit Trail system. Moreover, the system ensures that subjects are securely pseudonymised. All data hosted in the database is pseudonym, which means that the individual subject delivering the data entries cannot be identified without additional information. This information is stored exclusively on site. The investigator will be responsible for creating and securing the list of pseudonymization codes.

12.3 Monitoring

For quality control, the study site will be visited on-site by an appropriately trained and qualified monitor. All source data and relevant documents will be accessible to the monitor and questions will be answered during site visits. Any findings and comments will be documented in site visit reports and communicated to the Sponsor-Investigator.

- Site initiation visit: The visit will be done on site. The monitor will assess that all the study material and the infrastructure is ready to begin. The entire trial team at the site should be present.
- Monitoring visit: The first monitoring visit will take place within 2 weeks after the first inclusion. A monitoring visit will be scheduled for approximately every 10 patients enrolled during the inclusion phase if no major problems are observed. If needed, frequency of the monitoring visits can be increased. Informed consent form (ICF) controlled for 100% trial subject, 100% of the eCRF for 1st subject and for 10% of randomly selected patients, partial SVD (key data: eligibility, primary endpoint) for 100% for the subjects included, full review of the trial master file (TMF) at the beginning and the end of the study, security parameters (AE, SAE, device deficiencies) for 100% trial subject.
- Close out visit: Last visit before the closure of the study. It may be combined with the last on-site monitoring.

12.4 Audits and Inspections

In case of audits or inspections by the CEC or the CA, all source data and relevant documents will be accessible to the auditors/inspectors and questions will be answered during inspections. All involved parties must keep the subject data strictly confidential.

12.5 Confidentiality, Data Protection

Trial and participant data will be handled with extreme discretion and will only be accessible to authorized personnel who require the data to fulfil their duties within the scope of the study. On the eCRFs and other study-specific documents, participants will be identified by a unique participant number to ensure subject confidentiality.

The PI will be responsible for the secure storage of the subject identification list of the coded data for the trial. The subject identification will be kept in an electronic secure storage of the HUG during the course of the trial. The use of the secured eCRF of the secuTrial® platform hosted on HUG servers, will ensure the audit trail and secure authentication of the data.

Direct access to source documents will be permitted for purposes of monitoring of audits and inspections.

13. PUBLICATION AND DISSEMINATION POLICY

Results from the study will be published as one or more papers in international English language journals. Positive, negative and inconclusive results will be published. Study results ownership vested solely to the HUG. All rights to publication decisions remain with the scientific study partners (HUG and AAU). Authorship will be determined in the following order: Ulrike Pielmeier as first author, Aude de Watteville as second author, Steen Andreassen as second last author, and Claudia-Paula Heidegger as last author. The order of the other investigators will be determined according to their involvement during the conduct of the study.

14. FUNDING AND SUPPORT

14.1 Funding

This study is mainly funded by the Innovationsfonden, Danemark (72'968 CHF). In addition, we have obtained a grant from the European Society for Nutrition and Metabolism (ESPEN research grant (35,877 CHF)), a grant of Nestlé Health Science (37'000 CHF), a donation of Fresenius Kabi (20'000 CHF) and an institutional grant for the Department of Acute Medicine (50'000 CHF).

14.2 Other Support

Not applicable.

15. INSURANCE

Insurance will be provided by the Sponsor. The insurance contract will be received after approval of the study by the CEC and the CA.

16. REFERENCES

1. *Declaration of Helsinki*. 2013; Available from: http://www.wma.net/en/30publications/10policies/b3/index.html
2. *ISO 14155 Clinical investigation of medical devices for human subjects - Good clinical practice* 2011.
3. International Conference on Harmonization (ICH, *E6 Guideline for Good Clinical Practice. .* 1996.
4. *Ordonnance sur les essais cliniques de dispositifs médicaux (OClin-Dim).* 2020
5. *Medical Device Regulation (EU) 2017/745 of 5 April 2017 (MDR) (https://eur-lex.europa.eu/legal-content/EN/TXT/?uri=CELEX%3A32017R0745)*
6. *ISO 14971 Medical devices - Application of risk management to medical devices.* 2007.
7. *Loi fédérale relative à la recherche sur l'être humain (loi relative à la recherche sur l'être humain, LRH)* 2011.
8. *Ordonnance sur les essais cliniques hors essais cliniques de dispositifs médicaux (Oclin).* 2013.
9. Alberda, C., et al., *The relationship between nutritional intake and clinical outcomes in critically ill patients: results of an international multicenter observational study.* Intensive Care Med, 2009. **35**(10): p. 1728-37.
10. Preiser, J.C., et al., *Metabolic response to the stress of critical illness.* Br J Anaesth, 2014. **113**(6): p. 945-54.
11. Villet, S., et al., *Negative impact of hypocaloric feeding and energy balance on clinical outcome in ICU patients.* Clin Nutr, 2005. **24**(4): p. 502-9.
12. Kotagal, M., et al., *Perioperative hyperglycemia and risk of adverse events among patients with and without diabetes.* Ann Surg, 2015. **261**(1): p. 97-103.
13. Krinsley, J.S., *Glycemic control in the critically ill - 3 domains and diabetic status means one size does not fit all!* Crit Care, 2013. **17**(2): p. 131.
14. Clain, J., K. Ramar, and S.R. Surani, *Glucose control in critical care.* World J Diabetes, 2015. **6**(9): p. 1082-91.
15. Krinsley, J.S., *Association between hyperglycemia and increased hospital mortality in a heterogeneous population of critically ill patients.* Mayo Clin Proc, 2003. **78**(12): p. 1471-8.
16. Puthucheary, Z.A., et al., *Acute skeletal muscle wasting in critical illness.* JAMA, 2013. **310**(15): p. 1591-600.
17. Schefold, J.C., J. Bierbrauer, and S. Weber-Carstens, *Intensive care unit-acquired weakness (ICUAW) and muscle wasting in critically ill patients with severe sepsis and septic shock.* J Cachexia Sarcopenia Muscle, 2010. **1**(2): p. 147-157.
18. Wischmeyer, P.E., et al., *Muscle mass and physical recovery in ICU: innovations for targeting of nutrition and exercise.* Curr Opin Crit Care, 2017. **23**(4): p. 269-278.
19. Ali, N.A., et al., *Acquired weakness, handgrip strength, and mortality in critically ill patients.* Am J Respir Crit Care Med, 2008. **178**(3): p. 261-8.
20. Combes, A., et al., *Morbidity, mortality, and quality-of-life outcomes of patients requiring >or=14 days of mechanical ventilation.* Crit Care Med, 2003. **31**(5): p. 1373-81.
21. Wieske, L., et al., *Impact of ICU-acquired weakness on post-ICU physical functioning: a follow-up study.* Crit Care, 2015. **19**: p. 196.
22. Elke G., H.W.H., Kreymann K. G., Adolph M., Felbinger T.W., Graf T.,, H.A.R. de Heer G., Kampa U., Mayer K., Muhl E., Niemann B., , and S.S. Rümelin A., Stoppe C., Weimann A., Bischoff S.C., *DGEM Guideline "Clinical Nutrition in Critical Care Medicine".* Aktuel Ernahrungsmed, 2018(43): p. 341-408.
23. McClave, S.A., et al., *Guidelines for the Provision and Assessment of Nutrition Support Therapy in the Adult Critically Ill Patient: Society of Critical Care Medicine (SCCM) and American Society for Parenteral and Enteral Nutrition (A.S.P.E.N.).* JPEN J Parenter Enteral Nutr, 2016. **40**(2): p. 159-211.
24. Singer, P., et al., *ESPEN guideline on clinical nutrition in the intensive care unit.* Clin Nutr,

2019. **38**(1): p. 48-79.

25. Rood, E., et al., *Use of a computerized guideline for glucose regulation in the intensive care unit improved both guideline adherence and glucose regulation.* J Am Med Inform Assoc, 2005. **12**(2): p. 172-80.
26. Taylor, B.E., et al., *Efficacy and safety of an insulin infusion protocol in a surgical ICU.* J Am Coll Surg, 2006. **202**(1): p. 1-9.
27. Cahill, N.E., et al., *Nutrition therapy in the critical care setting: what is "best achievable" practice? An international multicenter observational study.* Crit Care Med, 2010. **38**(2): p. 395-401.
28. Chase, J.G., et al., *A glucose-insulin pharmacodynamic surface modeling validation and comparison of metabolic system models.* Biomedical Signal Processing and Control, 2009. **4**(4): p. 355-363.
29. Pielmeier, U., et al., *A simulation model of insulin saturation and glucose balance for glycemic control in ICU patients.* Comput Methods Programs Biomed, 2010. **97**(3): p. 211-22.
30. Pielmeier, U., et al., *Prediction Validation of Two Glycaemic Control Models in Critical Care*, in *International Federation of Automatic Control World Congress*. 2008: Seoul, Korea.
31. Pielmeier, U., et al., *Comparison of Identication Methods of a Time-varying Insulin Sensitivity Parameter in a Simulation Model of Glucose Metabolism in the Critically Ill*, in *IFAC Symposium on Modelling and Control in Biomedical Systems*. 2009: Aalborg, Denmark.
32. Pielmeier, U., et al., *The Glucosafe system for tight glycemic control in critical care: a pilot evaluation study.* J Crit Care, 2010. **25**(1): p. 97-104.
33. Pielmeier, U., et al., *Decision support for optimized blood glucose control and nutrition in a neurotrauma intensive care unit: preliminary results of clinical advice and prediction accuracy of the Glucosafe system.* J Clin Monit Comput, 2012. **26**(4): p. 319-28.
34. Riddersholm, S.J., et al., *Lowering of blood glucose and its variability by computerized decision support* Aalborg University, Center for Model-based Medical Decision Suppo.
35. Pielmeier, U., et al., *Comparison of Identication Methods of a Time-varying Insulin Sensitivity Parameter in a Simulation Model of Glucose Metabolism in the Critically Ill.* Proceedings of the 7th IFAC Symposium on Modelling and Control in Biomedical Systems , Aalborg, Denmark, August 12-14, 2009.
36. *MDCG 2020-10/1 Safety reporting in clinical investigations of medical devices under the Regulation (EU) 2017/745 (https://ec.europa.eu/health/sites/health/files/md_sector/docs/md_mdcg_2020-10-1_guidance_safety_reporting_en.pdf).*
